# Epigenetic Regulation of Intronic Transgenes in *Arabidopsis*

**DOI:** 10.1038/srep45166

**Published:** 2017-03-24

**Authors:** Kenji Osabe, Yoshiko Harukawa, Saori Miura, Hidetoshi Saze

**Affiliations:** 1 Plant Epigenetics Unit, Okinawa Institute of Science and Technology Graduate University, 1919-1 Tancha, Onna-son, Okinawa 904-0412, Japan

## Abstract

Defense mechanisms of plant genomes can epigenetically inactivate repetitive sequences and exogenous transgenes. Loss of mutant phenotypes in intronic T-DNA insertion lines by interaction with another T-DNA locus, termed T-DNA suppression, has been observed in *Arabidopsis thaliana*, although the molecular basis of establishment and maintenance of T-DNA suppression is poorly understood. Here we show that maintenance of T-DNA suppression requires heterochromatinisation of T-DNA sequences and the nuclear proteins, INCREASED IN BONSAI METHYLATION 2 (IBM2) and ENHANCED DOWNY MILDEW 2 (EDM2), which prevent ectopic 3′ end processing of mRNA in atypically long introns containing T-DNA sequences. Initiation of T-DNA suppression is mediated by the canonical RdDM pathway after hybridisation of two T-DNA strains, accompanied by DNA hypermethylation of T-DNA sequences in the F1 generation. Our results reveal the presence of a genome surveillance mechanism through genome hybridisation that masks repetitive DNAs intruding into transcription units.

Plants have evolved a genome defense system that can transcriptionally inactivate repetitive DNA, such as mobile transposable elements (TEs) and transgenes. The system involves epigenetic mechanisms, including inter-dependent modifications of RNA and chromatin such as small RNA production and DNA methylation, which facilitate long-term silencing of the “non-self” DNA sequences^1^. Molecular mechanisms to establish the transcriptionally silent chromatin state of these invading DNA elements have been a major focus of investigation.

In plants, repetitive DNAs are targeted and silenced by an RNA-based mechanism called RNA-directed DNA methylation (RdDM), which induces DNA methylation of a DNA template that leads to transcriptional gene silencing (TGS)^2^. Two pathways of RdDM have been described: RNA polymerase IV (PolIV)-RdDM and Polymerase II (PolII)-RNA-DEPENDENT RNA POLYMERASE 6 (RDR6)-dependent RdDM^3^. PolIV is recruited to loci associated with histone H3K9 methylation (H3K9me) and transcribes single-stranded RNA (ssRNA). The ssRNA becomes the template for 24-nucleotide (nt) small interfering RNA (siRNA) after being processed by RDR2 and DICER-LIKE 3 (DCL3). siRNA is loaded onto ARGONAUTE 4 (AGO4), which binds to non-coding scaffold RNA transcribed by RNA polymerase V (PolV). *NUCLEAR RNA POLYMERASE E1 (NRPE1*) encodes the largest subunit of PolV and *NUCLEAR RNA POLYMERASE D2a (NRPD2a*) encodes a shared subunit of RNA polymerase IV and V^2^. The chromatin remodeler DEFECTIVE IN RNA-DIRECTED DNA METHYLATION 1 (DRD1) is required for PolV activity. These factors recruit *de novo* methylase DOMAINS REARRANGED METHYLTRNANSFERASE 2 (DRM2) to methylate repeated DNA sequences. On the other hand, in the non-canonical PolII-RDR6-dependent RdDM pathway, PolII transcribed ssRNA from repeats is converted into double-stranded RNA (dsRNA) by RDR6, processed into 21–22nt siRNA by DCL2 and DCL4. The siRNA is loaded into AGO6, which can be directed to the scaffold RNA transcribed by PolV, establishing and reinforcing TGS. The DNA methylation established at CG and non-CG sites is maintained through cell divisions by DNA methylases METHYLTRANSFERASE 1 (MET1) and CHROMOMETHYLASEs (CMTs), and the chromatin remodeler DECREASE IN DNA METHYLATION 1 (DDM1)^1^,^4^.

Introduction of transgenes into plant genomes by agrobacterium-mediated transformation has been widely used for random mutagenesis of genomes, generation of transgenic plants conferring particular traits, and characterisation of genes of interest. A large collection of mutagenized plant lines, that have randomly integrated Transfer DNA (T-DNA) fragments containing exogenous DNA sequences such as viral promoters and bacterial antibiotics resistant genes, have been generated for model plants including *Arabidopsis thaliana*^5^. T-DNA inserted into exons is expected to produce transcripts interrupted by T-DNA sequences that do not code for the original protein, and intronic T-DNA insertions may also disrupt gene function by affecting proper transcription and splicing.

However, it has been well known that transgenes are often targeted by the host defense mechanisms. Particularly, introduction of a second, homologous T-DNA into the genome interferes with the other T-DNAs in *trans*, which is accompanied with DNA hypermethylation and gene silencing of promoters and antibiotic resistance genes encoded by T-DNA, a phenomenon called *trans*-inactivation^6^,^7^. *Trans*-inactivation can be induced by double-transformation of T-DNAs, or also by introduction of unlinked T-DNA by sexual crossing^8^,^9^. More recently, a phenomenon termed “T-DNA suppression” has been reported, in which genes remain functional despite the presence of T-DNA insertions within the introns of genes^10^,^11^,^12^. T-DNA suppression generally occurs after crossing two different T-DNA insertion mutants. There are several characteristic features of T-DNA suppression from these reports: (1) T-DNA suppression occurs after crossing two homologous T-DNA mutants (e.g., SALK T-DNA lines), (2) these mutants suppress one of the T-DNA mutant phenotypes, leaving the other mutant phenotype expressed, (3) it occurs in T-DNAs inserted into intronic regions, and (4) the suppressed state can be maintained for multiple generations in the absence of the second T-DNA that triggered the suppression. mRNA splicing machinery and DNA methylation seem to be associated with T-DNA suppression^11^,^12^. Splicing out of the intronic T-DNA and a high level of endogenous transcripts were observed in the *ben1-1 (BRI1-5 ENHANCED 1*) T-DNA suppressed line^11^. Similarly, a high transcript level was observed in *cob-6 (COBRA*) T-DNA suppressed lines^12^, and both of these T-DNA suppressed lines were associated with CG and CHG methylation. Furthermore, disruption of DNA demethylation activity by loss of REPRESSOR OF SILENCING1 (ROS1) function leads to T-DNA suppression of *cob-6* without the need of crossing with another T-DNA locus. Inhibition of DNA methylation by application of 5-azacytidine or zebularine, or loss of DNA methyltransferase activity resulted in the release of T-DNA suppression, re-acquiring the mutant phenotype^12^. Thus, epigenetic modulations of intronic T-DNA sequences have been suggested for T-DNA suppression, although detailed molecular mechanisms of induction, and maintenance of T-DNA suppression, as well as alteration of modes of gene transcription associated with suppression remain poorly understood.

This study investigated transcriptional changes in T-DNA suppressed lines and involvement of epigenetic pathways required to establish and maintain T-DNA suppression. T-DNA suppression is stably induced and maintained in intronic T-DNA mutants of the AGAMOUS (AG) and LEAFY (LFY) genes that contain >10 kb intron sequences containing T-DNA insertions. We demonstrated that T-DNA suppression promotes splicing of T-DNA-containing introns, which requires heterochromatinisation of T-DNA sequences, as well as the nuclear proteins, INCREASED BONSAI METHYLATION 2 (IBM2) and ENHANCED DOWNY MILDEW 2 (EDM2). Induction of T-DNA suppression is mediated by the canonical RdDM pathway, likely in the F1 generation after sexual crossing, which was associated with extensive DNA methylation of T-DNA sequences. Our results demonstrate the involvement of epigenetic mechanisms that can mask the influence of foreign DNA intruding into transcription units.

## Results

### Establishment of intronic T-DNA suppression in the presence of additional T-DNA

**Figure 1:**
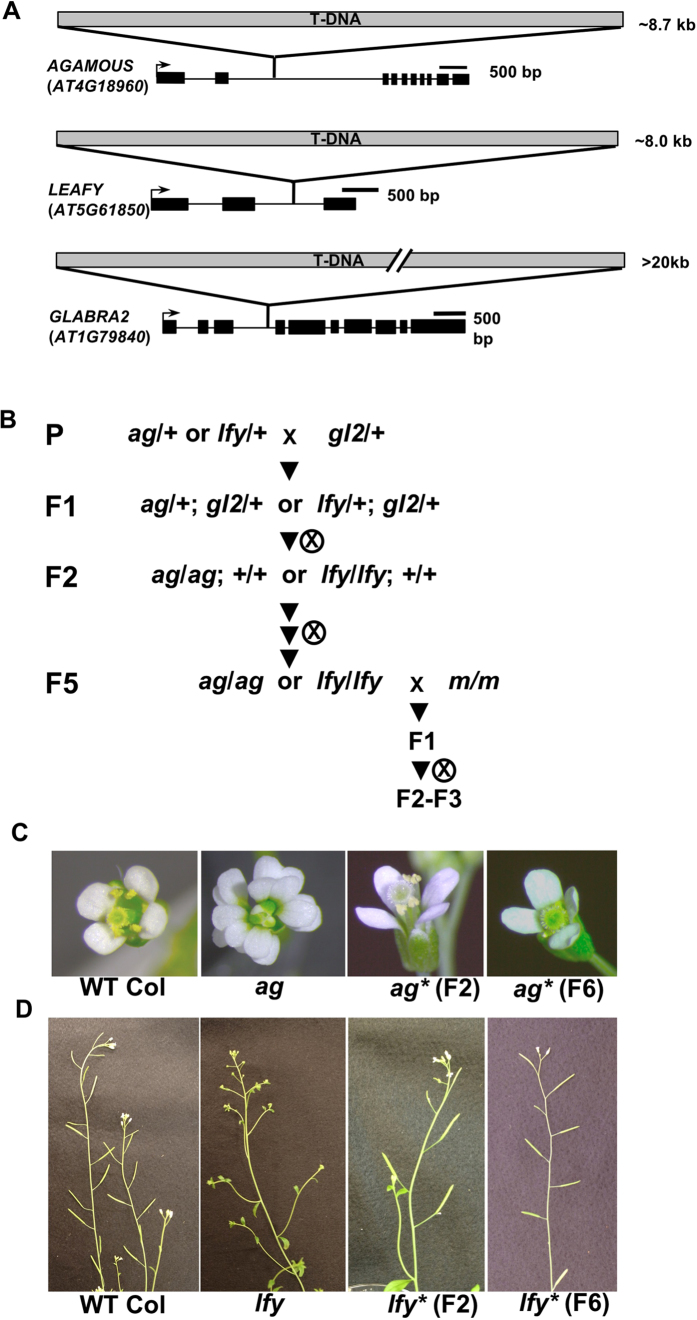
Suppression of intronic T-DNA in *Arabidopsis*. (**A**) Gene and T-DNA structure of *AGAMOUS, LEAFY*, and *GLABRA2* loci. Detailed structure determined by sequencing and Southern analyses are shown in Supplementary Figs S1–3. (**B**) The crossing scheme for induction of intronic T-DNA suppression. T4 segregants of heterozygous *ag, lfy* and *gl2* plants were used as parental lines for the crosses. “*m*” represents *met1, ddm1, cmt3, ibm2, edm2, nrpe1, nrpd2*, or *rdr6* (see also Fig. 2A,B). (**C**) Left to right: WT flower, a representative flower of an *ag* homozygous mutant, a WT-like flower on *ag* homozygote (ag*) in the F2, and F6 generations. (**D**) Left to right: WT inflorescence, a representative inflorescence of an *lfy* homozygous mutant, a WT-like inflorescence of an *lfy* homozygote (lfy*) in the F2, and F6 generations.

To investigate the molecular basis of epigenetic regulation of intronic transgenes, we selected the *Arabidopsis* SALK T-DNA lines, which have T-DNA insertions in genes such as *AGAMOUS, LEAFY*, and *GLABRA2*, mutants of which show visible phenotypes (Fig. 1A, Supplementary Figs S1–S3). *AGAMOUS (AG*) encodes a MADS domain transcription factor required for specification of stamen and carpel organs^13^. A previous study demonstrated that the T-DNA in SALK_014999 (*ag-12; ag* hereafter) inserted into the second intron of *AG* is suppressed in the presence of an additional T-DNA, and homozygous *ag* plants develop Wild-Type (WT)-like flowers^10^. *LEAFY (LFY*) is involved in floral meristem development, and *lfy* plant shows transformation of flowers into inflorescence shoots^14^, and SALK_057202 (*lfy* hereafter) has a T-DNA insertion in the second intron (Fig. 1A, Supplementary Fig. S2). *GLABRA2* encodes a homeodomain protein that regulates epidermal cell identity, including trichome formation^15^, and in the SALK_130213 (*gl2-8*; *gl2* hereafter) line, the T-DNA is inserted into the third intron of the gene (Fig. 1A, Supplementary Fig. S3). Southern analysis and DNA sequencing analysis of intronic T-DNA mutants showed that more than 8 kb of multi-copy T-DNAs and a part of the pROK2 binary vector sequence with complex rearrangements had been inserted at each locus, which extended the intron length longer than the original gene (Fig. 1A, Supplementary Figs S1–3).

Suppression of the *ag* phenotype was induced in the F2 generation by a cross between *ag* and *gl2*, as previously reported^10^ (Fig. 1B,C, Table 1). We also observed suppression of the *lfy* phenotype in the F2 when *lfy* was crossed with the *gl2* T-DNA line (Fig. 1B,D). In these crosses, approximately one-quarter to one-third of the F2 plants possessed homozygous T-DNA in *AG, LFY*, and *GL2*, as expected according to Mendelian segregation, but all of the F2 plants showed WT flower or inflorescence phenotypes (Table 1). These WT-like *ag* and *lfy* mutant plants containing homozygous T-DNA insertions were designated as suppressed T-DNA mutants ag* or lfy*, as previously described^10^. As reported^10^,^11^,^12^, suppressed *ag* and *lfy* phenotypes were stably inherited for at least five generations in the absence of the additional T-DNA in the *GL2* locus through self-pollination (Fig. 1C). However, we never observed suppression of the *gl2* phenotype in the F2 generation of these crosses, or in subsequent generations (Table 1, and data not shown). When *ag* and *lfy* were crossed, only the *lfy* phenotype was suppressed in the F2 generation (Table 1). Another T-DNA inserted in an intergenic region (SALK_ 095889) did not induce T-DNA suppression of *ag* and *lfy* (Table 1). We also found that allelic crosses, i.e. *ag* x *ag* or *lfy* x *lfy* did not induce T-DNA suppression as efficiently as crosses between T-DNA lines in *trans* (Table 1). On the other hand, the previous study showed that ag* can convert *ag* allele to ag*, showing a paramutation-like phenomenon^10^. We also observed a similar paramutagenic effect of the lfy* allele (Supplementary Fig. S4). These results suggested that not only the presence of additional T-DNA with sequence homology (i.e. SALK T-DNAs), but also the epigenetic state of the T-DNA might be important for the induction of T-DNA suppression.

### Intronic T-DNA suppression requires IBM2 and EDM2 to prevent ectopic 3′ end processing of introns

**Figure 2:**
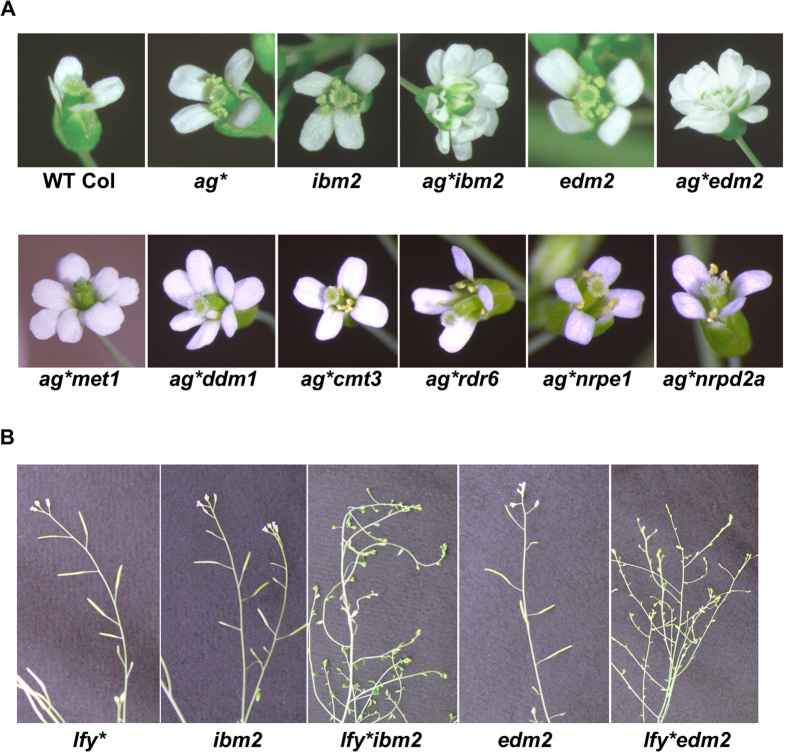
Epigenetic regulation of intronic T-DNA in *AG* and *LFY* loci. (**A**) *ag* flower phenotypes of plants in the F2 or F3 generations after crossing to various mutants (Fig. 1B). (**B**) Inflorescence phenotypes of F2 or F3 plants with indicated genotypes.

It has been suggested that epigenetic regulation is involved in suppression of intronic T-DNA^12^. To test the requirement of epigenetic factors in maintenance of the suppressed epigenetic state of intronic T-DNA, the ag* and lfy* lines were crossed with non-T-DNA mutants of maintenance DNA methylation, such as *met1, ddm1*, and *cmt3*, or of the RdDM pathway such as *nrpe1, nrpd2a*, and *rdr6*^2^,^3^ (Fig. 1B). In the F2 generation, we observed plants that regained the *ag* phenotype in the *met1* or *ddm1* backgrounds, while suppression did not require CMT3, nor RdDM factors for its maintenance (Fig. 2A). Recent studies demonstrated that efficient transcription and/or splicing of introns associated with heterochromatic epigenetic marks requires the nuclear proteins, IBM2 and EDM2^16^,^17^,^18^,^19^. IBM2 contains a Bromo-Adjacent Homology (BAH) domain and an RNA recognition motif (RRM), while EDM2 contains PHD finger domains that bind to H3K9 methylation. In the *ibm2* and *edm2* backgrounds, ag* and lfy* homozygous plants showed severe *ag* and *lfy* phenotypes that were not observed in *ibm2* or *edm2* single mutants, nor in the segregating *ag* and *lfy* homozygous siblings (Fig. 2A,B). As IBM2 and EDM2 likely act downstream of repressive epigenetic marks^20^, these results suggest that maintenance of T-DNA suppression requires a heterochromatic state maintained by MET1 and DDM1, and that IBM2 and EDM2 promote full-length transcription of *AG* and *LFY* over introns containing suppressed T-DNA sequences.

**Figure 3:**
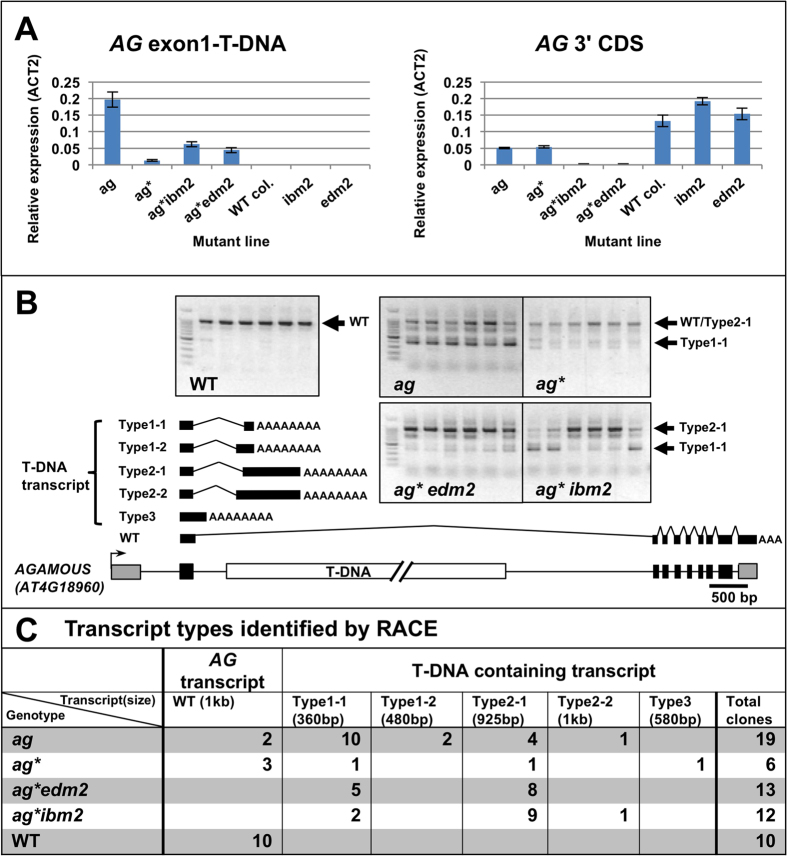
Transcription changes in suppressed *AG* intronic regions. (**A**) *AG* expression level measured by qRT-PCR. *ag;* parental *ag* mutant, ag*; suppressed *ag*, WT col.; non-transgenic Columbia. Bars are mean +/− SEM (n = 3). (**B**) Gel image of 3′-RACE. Five *AG* transcript types from WT, *ag*, and ag* T-DNA mutants were identified after cloning of the major bands. Type 3 transcripts retained intron 2 and the polyA-tail was found within the intron. All other transcript types have the same intron donor splice site as the endogenous *AG* intron 2, but have different acceptor sites within the T-DNA region. Transcript Types 1–2 and 2–2 have an alternative splice site 93 bp upstream of Types 1–1 and 2–1. Six independent plants were examined for each genotype. Gray and black boxes in the AG gene structure represent UTRs and exons, respectively. (**C**) Transcript types identified by sequencing RACE products. Both *ag* and ag* contained a mix of WT and Type 2–1 transcripts in the predominant upper band at 1 kb, but *ag* also produced another predominant band of Type 1–1 transcripts. Total numbers of clones identified are indicated.

Transcript analysis of *AG* before and after induction of T-DNA suppression showed that parental *ag* plants accumulate transcripts in the region upstream of the T-DNA insertion (exon1), while the transcript level in the 3′ end of the *AG* coding sequence (CDS: exon 8–9) downstream of T-DNA insertion decreased, compared to wild-type (Fig. 3A). 3′-RACE demonstrated that plants with the *ag* phenotype accumulated shorter transcripts containing part of the T-DNA sequence, and were prematurely polyadenylated before or within T-DNA insertion sites (Fig. 3B, Supplementary Fig. S5A). After induction of T-DNA suppression, ag* showed decreased levels of shorter transcripts terminated within the T-DNA relative to *ag* (Fig. 3A,B), but expressed more WT transcripts (Fig. 3C). When ag* was crossed into the *ibm2* and *edm2* backgrounds, the 3′ end of *AG* expression was decreased due to premature termination of the transcript within the T-DNA region (Fig. 3A,B, Supplementary Fig. S5B). These data suggest that T-DNA suppression in *AG* and *LFY* loci is due to enhanced splicing of T-DNA-containing introns and that it requires *IBM2* and *EDM2* to prevent ectopic 3′ end processing of the long introns containing T-DNA sequences.

### DNA methylation covers the entire sequence of T-DNA in the suppressed state

**Figure 4:**
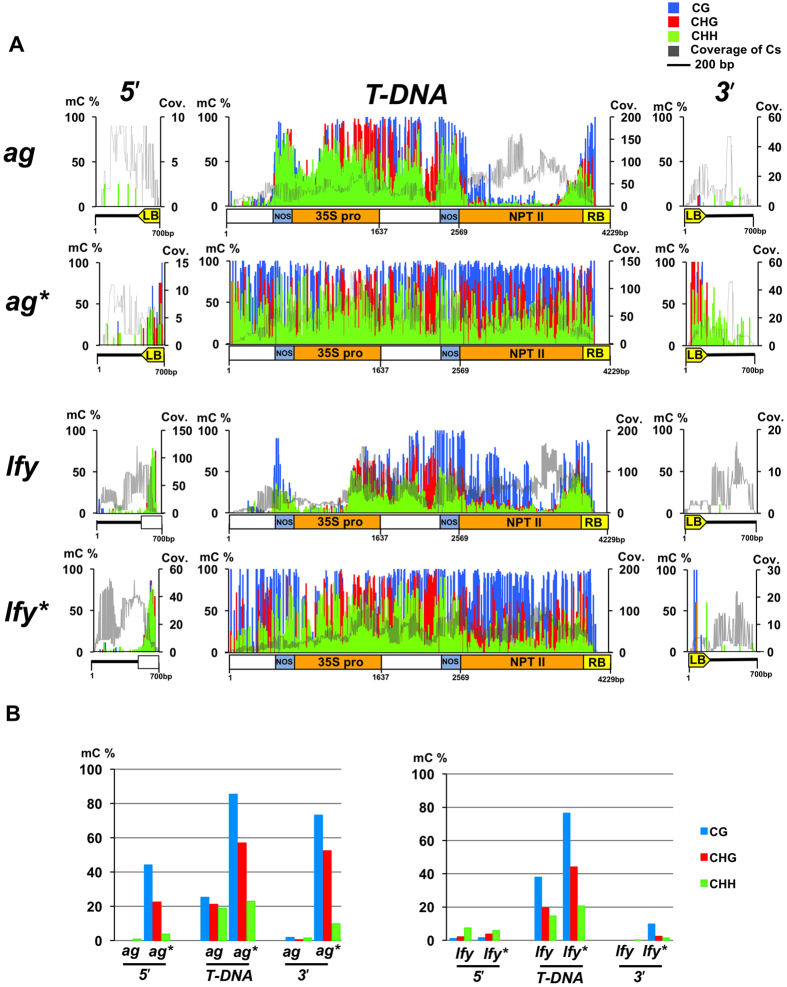
BS-seq analyses of the 5′ and 3′ borders of the T-DNA insertion site and flanking intron sequences, as well as T-DNA regions in *ag* and *lfy* single mutants. (**A**) A graphical representation of DNA methylation (CG, CHG, and CHH) status of representative samples with indicated genotypes/epigenotypes. *ag* and *lfy* represent DNA methylation in T-DNA regions of T4 homozygous plants before suppression. For analysis of ag* and lfy*, genomic DNA from F7 plants was used. Gray lines represent the number of cytosines covered by BS-seq reads. Cytosines covered by fewer than 4 reads were excluded from the analysis. (**B**) A summary of DNA methylation analysed in (**A**).

To know whether the suppression is associated with epigenetic changes in intronic T-DNA, we examined DNA methylation in T-DNA regions with Bisulfite-sequencing (BS-seq) analysis. Single homozygous mutants of *ag, lfy*, and *gl2* plants before and after induction of suppression were used to avoid mixing sequencing reads originating from multiple loci. Still, most of the sequence reads could be mapped to multiple regions, since multi-copy T-DNA sequences are inserted in intronic regions of *AG, LFY*, and *GL2* loci (Supplementary Figs S1–3). Therefore, sequence reads were mapped to the original T-DNA region of the pROK2 vector sequence, including unique flanking regions of T-DNA insertion sites, in order to determine the average DNA methylation level over the T-DNA sequences. We found that even before induction of suppression, a large proportion of T-DNA sequences was highly methylated at both CG and non-CG sites, while still some regions remain unmethylated (Fig. 4A,B). However, suppressed T-DNAs showed hypermethylation at CG and non-CG sites throughout the sequences. Considering that an average of 85% (ag*) and 76% (lfy*) of CG methylation is induced after suppression, methylation is likely distributed evenly across multi-copy sequences of T-DNAs (Supplementary Figs S1–2). Especially, 5′ regions of T-DNA, where premature termination of transcripts was observed (Fig. 3), was fully covered by DNA methylation in ag* and lfy* compared to non-suppressed plants (Fig. 4). In addition, DNA methylation was spread into the flanking regions of the T-DNA sequences in ag* and lfy* lines. These results were consistent with data obtained from McrBC-PCR and Bisulfite-PCR analyses for the 5′ flanking region of T-DNAs (Supplementary Figs S6–S8). Interestingly, the DNA methylation pattern at the T-DNA sequence in the *GL2* locus, which never showed suppression of the phenotype, was largely unchanged after crossing (Supplementary Fig. S9). No large changes in DNA methylation were observed after crossing ag* with *ibm2*, or *edm2* (Supplementary Figs S7,S8), consistent with previous data that *ibm2* and *edm2* affect processing of heterochromatic introns without changes in DNA methylation^18^,^20^. DNA methylation is comparable between ag* and ag*met1 at the 5′ ends of T-DNA sequences (Supplementary Fig. S7), while methylation, especially in CG context, was reduced in the 35S promoter sequence(s) of T-DNA in ag*met1 and ag*ddm1 (Supplementary Fig. S8). These data suggest that DNA methylation of the entire T-DNA sequence, including the 5′ and 3′ borders, might be required for T-DNA suppression.

### Establishment of intronic T-DNA suppression requires RdDM factors

**Figure 5:**
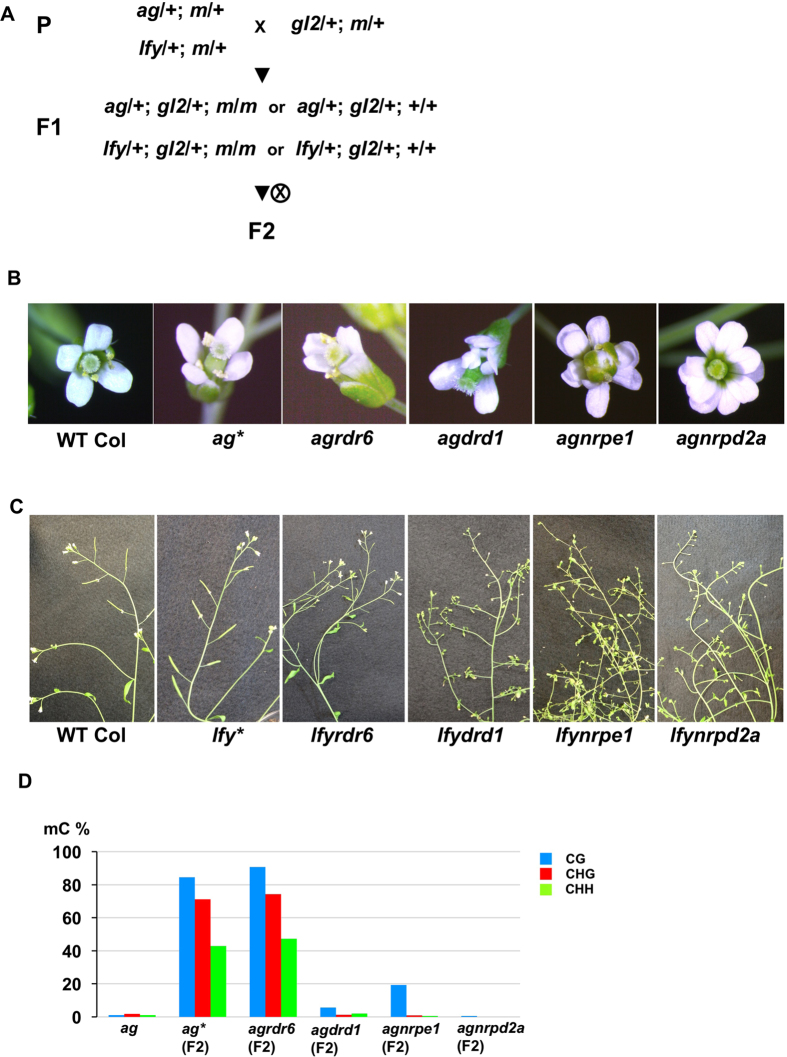
Establishment of T-DNA suppression in various mutant backgrounds. (**A)** Cross scheme of T-DNA mutants and mutants of factors involved in the RNA-directed DNA methylation pathway. “*m*” represents *rdr6, drd1, nrpe1*, or *nrpd2a*. (**B**) Flowers of F2 plants with the indicated genotype obtained by the cross shown in (**A**). (**C**) Inflorescence of F2 plants with the indicated genotype obtained by the cross shown in (**A**). (**D**) A summary of DNA methylation in the 5′ region of T-DNA in *AG* locus in the plants with the indicated genotype. See also Fig. S11.

**Figure 6:**
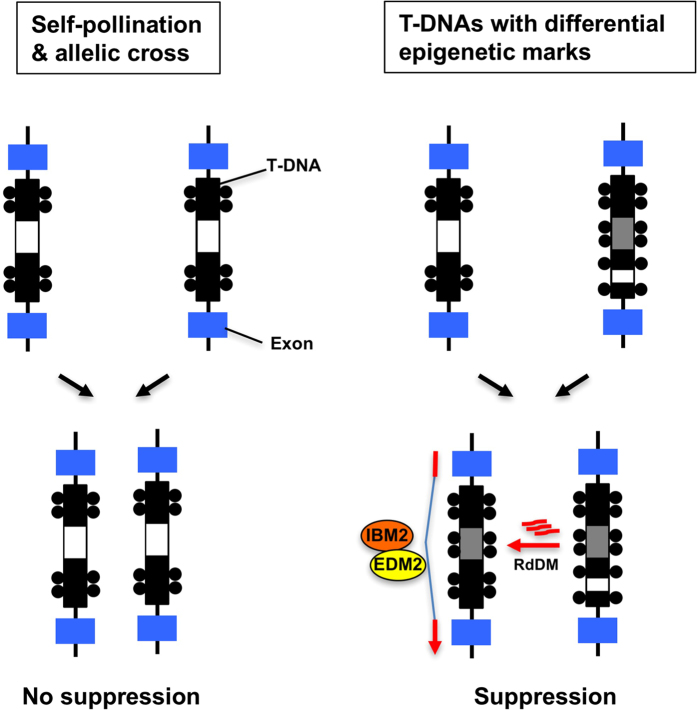
A model for establishment and maintenance of epigenetic suppression of intronic T-DNA. Black circles represent DNA methylation. Black/gray and blue boxes represent methylated T-DNA sequence, and exons, respectively. White boxes within the T-DNA represent unmethylated regions.

We further analysed how T-DNA suppression is epigenetically established. We found that hypermethylation of *ag* T-DNA in the 5′ flanking sequence had occurred in the F1 generation (Supplementary Fig. S10). The 5′ flanking sequence of *lfy* T-DNA was already methylated before suppression, but further methylated especially in non-CG contexts in the F1 generation after crossing with *gl2* (Supplementary Fig. S10). The T-DNA inserted in *GL2* was already highly methylated in the parental line, which remained largely unchanged in the F1 and F2 generations. These data suggest that crossing of two different T-DNA lines induces further DNA methylation in suppressed T-DNA sequences in the F1 generation. To test whether DNA methylation mediated by RdDM factors is required for establishment of T-DNA suppression, the *ag* and *lfy* T-DNA lines were crossed with the *gl2* T-DNA line in the absence of RdDM factors in the F1 generation (Fig. 5A). To avoid effects of additional T-DNA sequences, point mutants of RdDM genes were used. When *NRPE1, DRD1*, and *NRPD2a* were mutated in the F1 generation, suppression of *ag* and *lfy* was not observed in the F2 generation (Fig. 5B,C), where plants with *ag* or *lfy* phenotypes segregated in an approximately 3:1 ratio (Table 2). The appearance of the *ag* phenotype is consistent with the absence of DNA methylation in the T-DNA sequence (Fig. 5D, Supplementary Fig. S11). This result is in clear contrast to double mutants of ag* and RdDM factors (Fig. 2A). In contrast, *RDR6* was not required for the establishment of T-DNA suppression (Fig. 5D, Supplementary Fig. S11), suggesting that canonical RdDM factors are responsible for establishment of T-DNA suppression (Fig. 6).

## Discussion

In this study, T-DNA suppression was efficiently induced in a cross between *ag* or *lfy* and *gl2* SALK T-DNA mutants, where F2 progeny were all WT phenotype, despite the presence of homozygous intronic T-DNA insertions in the AG and LFY genes. The production of suppressed T-DNA mutants was reproducible and the effect was stable for at least five generations, which allowed us to use this system to investigate how T-DNA suppression is established and maintained, by crossing mutants of genes involved in known epigenetic pathways.

Suppression of intronic T-DNA and loss of *ag* or *lfy* phenotypes from mutant plants suggested that in suppressed plants, introns with T-DNA are efficiently and stably spliced out (Figs 1 and 3). The intron harboring T-DNA in *AG* is about 12 kb in length (Fig. 1A). *Arabidopsis* genes contain relatively short introns (~160 bp in average) compared with genes in other plant species (~390 bp in rice, ~510 bp in maize)^21^, due to less abundant repeats in intronic regions^20^,^22^,^23^. According to the *Arabidopsis* TAIR10 annotation, the putative longest intron in the genome is about 11 kb, encoded in *AT2G34100*^24^. However, a previous report showed that at least 17 kb intronic region in the OPR3 gene, containing a T-DNA insertion, can be transcribed and spliced out under biotic stress condition^25^. This suggests that plant PolII transcription and splicing machineries have the potential to transcribe and splice these irregularly long introns. We never observed suppression of the T-DNA insertion in the intron of the GL2 gene, which might be due to the generation of an exceptionally long intronic region (>20 kb) that could exceed the capacities of Pol II transcription and/or splicing machineries (Fig. 1A, Supplementary Fig. S3). However, PolII has the potential to transcribe much longer genes in the genomes of other organisms. For example, the human dystrophin gene is the longest known gene in the human genome that is 2.2 Mb long with introns of over 100 kb, which is transcribed by Pol II and co-transcriptionally spliced^26^. This suggests that in addition to the intron length, there may be other factors required for stable establishment of T-DNA suppression.

Introduction of *ibm2, edm2, met1*, and *ddm1* mutations in suppressed ag* allowed to recover the *ag* mutant phenotype (Fig. 2). *IBM2* and *EDM2* are genes likely involved in 3′ end processing of mRNA transcribed over the heterochromatic intron^16^,^17^,^20^, suggesting that T-DNA suppression requires a heterochromatic region to splice out the T-DNA-containing intron. How IBM2 specifically recognizes heterochromatin in intronic regions is still not clear. On the other hand, EDM2 can bind to heterochromatin associated with T-DNA sequences via PhD domains^27^. MET1 and DDM1 are involved in DNA methylation and heterochromatin maintenance^28^,^29^, and MET1 has previously been shown to be required for T-DNA suppression^12^. Loss of heterochromatin may prevent IBM2 and EDM2 interaction with intronic regions, ultimately losing the ability to produce a full-length pre-mRNA and splice out the T-DNA-containing introns for production of functional transcripts. This supports the strong relationship of DNA methylation and T-DNA suppression, and the involvement of heterochromatin and splicing machinery to regulate T-DNA-containing introns.

DNA methylation of splice sites has been reported to influence splicing in maize and bees^30^,^31^. We observed an increase of DNA methylation in both 5′ and 3′ border regions, as well as inside T-DNA sequences in the suppressed lines (Fig. 4, Supplementary Figs S6–9). Previous studies have shown that 35S promoters in T-DNAs cause *trans*-inactivation between homologous sequences^6^. In our study, 35S promoter region(s) of T-DNAs in *AG* and *GL2* were already highly methylated before crossing (Figs 4, S8), suggesting that the homology of T-DNA sequences or the presence of hypermethylated 35S promoter sequences may be required, but not sufficient for the induction of intronic T-DNA suppression.

Transcript analysis of *ag* and ag* showed distinct expression patterns between mutants that exhibited the *ag* phenotype (ag, ag*ibm2 and ag*edm2) and ag* WT-like phenotype (Fig. 3). In general, the ratio of transcripts from upstream to those from downstream of the T-DNA is high in plants showing the *ag* phenotype, whereas the ratio is low in plants with WT-like flowers (Fig. 3A). Five different transcript isoforms of *AG* mRNA were identified in *ag* homozygous plants, and the *ag* mutant phenotype is associated with transcripts containing the 5′ region of the T-DNA sequence. T-DNA insertion creates an alternative splice acceptor site and ectopic polyadenylation sites that terminate the transcript within the T-DNA, leading to accumulation of non-functional transcripts (Fig. 3, Supplementary Fig. S5). Higher levels of WT transcripts in ag* and T-DNA suppression correlated well (Fig. 3), demonstrating that T-DNA suppression is a result of re-acquiring functional transcripts by splicing out T-DNA-containing introns. The level of WT *AG* transcripts from ag* is relatively low compared to controls, but may be sufficient for normal flower development, as shown by the lack of mutant phenotypes. Loss of IBM2 or EDM2 in ag* led to a significant loss of full-length *AG* transcripts, which explains the reappearance of the mutant phenotype.

The first interaction between the two T-DNA loci occurs when the parental genomes merge during fertilisation, and likely establishes T-DNA suppression in the F1 generation. Since *ag* and *lfy* heterozygous plants do not show suppression of the phenotypes after either crossing with WT or after self-pollination, the mechanism of T-DNA suppression is different from that of silencing unpaired sequences during meiosis^32^. Although AG, LFY, and GL2 genes are concomitantly expressed in the shoot apex^33^, whether T-DNA suppression requires co-transcription of T-DNA containing introns in the same tissue is not clear. T-DNA suppression in F2 progeny was not observed when *NRPD2a, NRPE1*, or *DRD1* are mutated in F1 plants (Fig. 5, Table 2, Supplementary Fig. S11). These genes are required for PolV-mediated *de novo* methylation and transcriptional gene silencing of TEs^2^. It has been reported that PolII-transcribed TE RNAs can enter the Pol II-RDR6-dependent RdDM pathway^3^,^34^. However, the *RDR6* mutation did not inhibit T-DNA suppression in our system (Fig. 5, Table 2, Supplementary Fig. S11). It is still possible that the Pol II-transcript from T-DNA-containing introns form a dsRNA hairpin structure (Supplementary Figs S1–S3), resembling the dsRNA transgene system triggering RdDM in *trans*^35^, which could be directly processed by DCL3 into 24nt siRNAs^36^. Another scenario is that PolIV-dependent 24nt siRNAs may already be generated from hypermethylated regions of the T-DNA before crossing, which may act as a *trans* signal to induce RdDM on the homologous sequence. The differential epigenetic composition of each T-DNA sequence (Fig. 4, Supplementary Fig. S9) may explain the inefficiency of suppression in self-pollination or allelic T-DNA crosses (Table 1), since in such cases, siRNAs required for induction of *de novo* methylation at unmethylated regions may not be available. In contrast, T-DNAs that can efficiently induce suppression *in trans* (e.g. the T-DNA in *GL2* locus) may generate siRNAs corresponding to unmethylated regions in the other T-DNA sequence, which trigger *de novo* methylation to fill “methylation gaps” for induction of suppression (Fig. 6).

Epigenetic alterations between two homologous sequences in F1 hybrids after intra- and interspecific hybridization have been described in many plant species^37^,^38^,^39^. In hybrid plants, DNA methylation and histone modifications on one chromosome can be transferred to other homologous regions, likely via siRNAs, which can sometimes induce heritable changes of gene expression and phenotypes^40^,^41^,^42^. Paramutation is one such well-known phenomenon, where allelic transfer of epigenetic states occurs in hybrid plants^43^, and indeed paramutation-like effects have been observed between two T-DNA sequences in *Arabidopsis* F1 plants^12^,^44^. In this study, we also observed a paramutagenic effect of lfy* allele, which can convert *lfy* to lfy* (Supplementary Fig. S4).

An intriguing observation in this study is the unidirectional induction of suppression. The T-DNA in *GL2* can suppress T-DNAs in *AG* and *LFY* loci but not vice versa, and when *ag* and *lfy* were crossed, only the T-DNA in *LFY* was suppressed (Table 1). The direction of T-DNA suppression between the T-DNA mutants may arise from the differences in epigenetic states between the T-DNA inducing suppression and the T-DNA being suppressed. This may be further complicated by the structures of the T-DNA integrated in each locus. The T-DNA in *LFY* locus show relatively high DNA methylation in both 5′ border of the T-DNA and the flanking intron sequence, even before suppression (Fig. 4), and often show spontaneous suppression (Table 1) that may explain why the LFY T-DNA suppression is facilitated by other T-DNAs. On the other hand, ag* and lfy* can induce suppression to their homologous, non-suppressed T-DNA alleles^10^ (Fig. S4), suggesting that the epigenetic state of “suppressed” T-DNAs (i.e. ag* and lfy*), such as DNA methylation and production of siRNAs, also causes distinct responses in non-suppressed T-DNAs. The T-DNA in the *GL2* locus is never suppressed by the other T-DNAs tested (Table 1), perhaps because the T-DNA sequence is too long to be suppressed by epigenetic mechanisms, or because it forms structures too divergent from the original T-DNA structure, which is not complementary to the siRNAs produced from other T-DNA loci. Dominance/recessive relationships among T-DNA loci are analogous to the self-incompatibility system in *Brassica* species, where small RNAs produced from dominant S haplotypes epigenetically suppress recessive S haplotypes^45^.

Inactivation of unlinked homologous T-DNA sequences after hybridization resembles to RIP (Repeat-induced point mutation) and MIP (Methylation induced premeiotically) in fungi, which are important genome surveillance mechanisms to detect duplication of DNA sequences and transposition of TEs^46^. Indeed, a recent report demonstrated that *de novo* TE insertion in an intronic region is suppressed after sexual crossing, likely through interactions with endogenous TE copies in other loci^47^. The paramutation-like effect may also allow a quick suppression of intronic TEs within the population. The experimental system employed in this study, which reproduces stable *trans*-inactivation of homologous T-DNA sequences should be useful to decipher the molecular basis of diverse epigenetic phenomena, and should advance our understanding of agriculturally important traits, such as self-incompatibility, sex determination, and heterosis^45^,^48^,^49^.

## Materials and Methods

### Plant materials

T-DNA insertion lines of *AGAMOUS* (SALK_014999; AT4G18960), *LEAFY* (SALK_057202; AT5G61850), *GLABRA2* (SALK_130213; AT1G79840), and SALK_095889 were obtained from the *Arabidopsis* Biological Stock Center^5^ (https://abrc.osu.edu). *ddm1-1, met1-1, cmt3-i11, rdr6-11*, and *ibm2-1* were described previously^16^,^50^,^51^,^52^,^53^. Seeds of *drd1-9, nrpe1-7, nrpd2a-7*, kindly provided by Dr. Tatsuo Kanno, were described previously^54^,^55^. The T4 segregants of heterozygous *ag, lfy* and *gl2* plants were used as parental lines for the crosses in Fig. 1B. An allele of *ENHANCED DOWNY MILDEW2 (EDM2*)^56^ (designated *edm2-9*), was isolated from the genetic screen described previously^57^, which has a G to A transition in the splice acceptor site at 4,553-bp downstream from the ATG translation start site of the EDM2 gene. All primers used in this study are listed in Supplementary Table 1.

PCR conditions for *AG* and *LFY* loci: (1) 95 °C for 2 minutes, (2) 95 °C for 20 seconds, (3) 52 °C for 30 seconds. (4) 72° for 1 minute, (5) 72 °C for 7 min. For the *GL2* locus: (1) 95 °C for 2 minutes, (2) 95 °C for 20 seconds, (3) 57 °C for 30 seconds. (4) 72° for 1 minute, (5) 72 °C for 7 min.

### RNA/DNA analyses

Genomic DNA (gDNA) was isolated using a DNeasy Plant Mini Kit (QIAGEN) or a Maxwell 16 LEV Plant DNA kit (Promega Corporation, USA), following manufacturers’ instructions. Total RNA was isolated using an RNeasy Plant Mini Kit (QIAGEN) or a Maxwell 16 LEV Plat RNA kit (Promega) according to kit instructions.

For qRT-PCR, cDNA was synthesized using 1–2 μg of total RNA and Prime Script II (TAKARA) with oligo-dT or random hexamers, following the supplier protocols. qRT-PCR was performed using KAPA Universal SYBR Mix (KAPA Biosystems) and cDNA that was diluted 5 to 10-fold. All reactions were performed in duplicate.

DNA methylation was examined by quantifying the amount of gDNA after treating it with the McrBC enzyme (TAKARA), which recognizes and cleaves methylcytosine-containing DNA. 200 ng of gDNA was digested with 2 Units of McrBC enzyme, and equal amounts of gDNA were mock treated as controls. McrBC- and mock-treated samples were diluted 10-fold in water. qPCR was performed in duplicate according to manufacturer instructions, using 4 μL of diluted McrBC- or mock-treated samples using KAPA Universal SYBR mix (KAPA Biosystems).

For 3′-RACE analysis, cDNA was synthesized using 1 μg of total RNA, oligo-dT T7 2–3 primer, which contains an adaptor sequence (Supplementary Table 1), and Prime Script II 1st strand cDNA synthesis kit (TAKARA) following the manufacturer’s protocol. 3′-RACE products were amplified with two rounds of touch-down PCR amplification. PCR conditions for both rounds were: (1) 95 °C for 2 minutes, (2) 95 °C for 20 seconds, (3) 65 °C for 30 seconds. (4) 72° for 1 minute, (5) repeat steps 2–4 for nine cycles decreasing 1 °C/cycle at step 3, (6) 95 °C for 20 seconds, (7) 55 °C for 30 seconds, (8) 72 °C for 1 minute, (9) repeating steps 6–8 for 25 cycles, and (10) 72 °C for 7 min. First amplification was performed using 2 μL of undiluted cDNA using HotStar PCR kit (QIAGEN). The first PCR product was diluted 10-fold and used for the second round of PCR. PCR products were cloned into pGEM-T Easy vector (Promega) and transformed into ECOS *E. coli* DH5-α (Nippon Gene) and sequenced. Sequences of T-DNA inserted into introns of *AGAMOUS, LEAFY*, and *GLABRA2* were determined using Universal GenomeWalkerTM 2.0 (Clontech Laboratories) following the manufacturer’s instructions.

### Bisulfite sequencing analysis

Bisulfite conversion of gDNA (0.4–1.0 μg) was performed as previously described^16^ or using EZ DNA Methylation-Gold kit (Zymo Research Corporation, USA) following the manufacturer’s instructions. BS-PCR was performed using Ex Taq DNA polymerase (TAKARA) or Go Taq Master Mix (Promega). PCR products were ligated into pGEM-T Easy vector and sequenced as above.

For Bisulfite-sequencing (BS-seq) analysis, we used gDNAs of *ag, lfy*, and *gl2* homozygous plants before crossing obtained from T4 populations, and ag* (F7), lfy*(F7) plants showing suppressed phenotypes without the second T-DNA that triggered the suppression. gDNA of un-suppressed *gl2* homozygous plants was obtained in an F2 segregating population by crossing it with an *ag* heterozygous plant. The absence of additional T-DNAs in the genome was confirmed by southern hybridization analysis (Supplementary Figs S1–S3) and PCR. An Illumina Sequencing library (180-bp pair-end) was constructed using the PBAT method^58^ and sequenced by the OIST Sequencing Center. Reads were mapped to the T-DNA region of the pROK vector sequence (TAIR accession Vector: 4775608)^59^, and to the flanking genome sequences of T-DNA insertion sites, including 200 bp of the left-border sequence, using Bismark^60^. Cytosine bases covered by fewer than 4 reads were excluded from the analysis.

### Southern analysis

Two micrograms of gDNA were digested with either *EcoR*I or *Hind*III, resolved on 1% Tris-Acetate-EDTA (TAE) gels, and blotted on Hybond N+ membranes (GE Healthcare Life Science). DNA probes corresponding to the sequences of left-border or the Neomycin phosphotransferase II (NPT II) gene in the pROK2 vector were amplified with PCR. Labeling of the probes and hybridisation were performed with Gene Images AlkPhos Direct Labeling and Detection System (GE Healthcare Life Science) following the manufacturer’s instructions. Chemifluorescence was detected with LAS-3000 (GE Healthcare Life Science).

## Additional Information

**Accession codes:** Sequencing data have been deposited in the DDBJ Sequence Read Archive under accession code DRA005188.

**How to cite this article:** Osabe, K. *et al*. Epigenetic Regulation of Intronic Transgenes in *Arabidopsis.* *Sci. Rep.* **7**, 45166; doi: 10.1038/srep45166 (2017).

**Publisher's note:** Springer Nature remains neutral with regard to jurisdictional claims in published maps and institutional affiliations.

## Supplementary Material

Supplementary Figures

## Figures and Tables

**Table 1:** T-DNA suppression observed in corsses of SALK T-DNA lines.

Cross		F1 genotype					*n*	*p*-value*
♀	♂		F2 genotype		F2 phenotype			
*ag*/+	*gl2*/+	*ag*/+; *gl2*/+	+/+, *ag*/+: 66	*ag*/*ag*: 30	WT: 96	*ag*: 0	96	1.54E-08
			+/+, *gl2*/+: 73	*gl2*/*gl2*: 23	WT: 73	*gl2*: 23	96	0.813
*gl2*/+	*ag*/+	*ag*/+; *gl2*/+	+/+, *ag*/+: 36	*ag*/*ag*: 11	WT: 47	*ag*: 0	47	7.55E-05
			+/+, *gl2*/+: 40	*gl2*/*gl2*: 7	WT: 40	*gl2*: 7	47	0.109
*ag*/+	+/+	*ag*/+	—	—	WT: 36	*ag*: 12	48	1
+/+	*ag*/+	*ag*/+	—	—	WT: 38	*ag*: 10	48	0.504
*lfy*/+	*gl2*/+	*lfy*/+; *gl2*/+	+/+, *lfy*/+: 32	*lfy*/*lfy*: 16	WT: 48	*lfy*: 0	48	6.33E-05
			+/+, *gl2*/+: 37	*gl2*/*gl2*: 11	WT: 37	*gl2*: 11	48	0.738
*gl2*/+	*lfy*/+	*lfy*/+; *gl2*/+	+/+, *lfy*/+: 52	*lfy*/*lfy*: 20	WT: 72	*lfy*: 0	72	9.63E-07
			+/+, *gl2*/+: 49	*gl2*/*gl2*: 23	WT: 49	*gl2*: 23	72	0.173
*lfy*/+	+/+	*lfy*/+	—	—	WT: 35	*lfy*: 12	47	0.9328853
+/+	*lfy*/+	*lfy*/+	—	—	WT: 45	*lfy*: 6	51	0.029
*lfy*/+	*ag*/+	*lfy*/+; *ag*/+	—	—	WT: 37	*ag*: 9	46	0.394
			—	—	WT: 46	*lfy*: 0	46	9.01E-05
*ag*/+	*lfy*/+	*lfy*/+; *ag*/+	—	—	WT: 39	*ag*: 9	48	0.317
			—	—	WT: 48	*lfy*: 0	48	6.33E-05
SALK_095889	*ag*/+	*ag*/+; SALK_095889/+	—	—	WT: 33	*ag*: 15	48	0.317
SALK_095889	*lfy* /+	*lfy* /+; SALK_095889/+	—	—	WT: 34	*lfy*: 14	48	0.505
♀	♂		F1 genotype		F1 phenotype		*n*	
*ag*/+	*ag*/+		+/+, *ag*/+: 16	*ag*/*ag*: 3	WT: 16	*ag*: 3	19	0.353
*lfy*/+	*lfy*/+		+/+, *lfy*/+: 20	*lfy*/*lfy*: 5	WT: 20	*lfy*: 5**	25	0.563

*Chi-square test with the expectation of 3:1 segregation ratio.

**Inflorescences often showed spontaneous changes to WT-like phenotype.

**Table 2:** Requirement of RNA-directed DNA methylation factors for T-DNA suppression.

Cross		F1 genotype			*n*	*p*-value*
♀	♂		F2 phenotype			
*ag*/+; *nrpe1*/+	*gl2*/+; *nrpe1*/+	*ag*/+; *gl2*/+; *nrpe1*/*nrpe1*	WT: 46	*ag*: 8	54	6.46E-03
		*ag*/+; *gl2*/+; +/+	WT: 48	*ag*: 0	48	
*lfy*/+; *nrpe1*/+	*gl2*/+; *nrpe1*/+	*lfy*/+; *gl2*/+; *nrpe1*/*nrpe1*	WT: 39	*lfy*: 6	45	6.04E-03
		*lfy*/+; *gl2*/+; +/+	WT: 57	*lfy*: 0	57	
*ag*/+; *drd1*/+	*gl2*/+; *drd1*/+	*ag*/+; *gl2*/+; *drd1*/*drd1*	WT: 53	*ag*: 6	59	2.60E-02
		*ag*/+; *gl2*/+; +/+	WT: 56	*ag*: 0	56	
*lfy*/+; *drd1*/+	*gl2*/+; *drd1*/+	*lfy*/+; *gl2*/+; *drd1*/*drd1*	WT: 32	*lfy*: 8	40	8.26E-04
		*lfy*/+; *gl2*/+; +/+	WT: 52	*lfy*: 0	52	
*ag*/+; *nrpd2a*/+	*gl2*/+; *nrpd2a*/+	*ag*/+; *gl2*/+; *nrpd2a*/*nrpd2a*	WT: 37	*ag*: 11	48	N.A.
		—	—	—		
*lfy*/+; *nrpd2a*/+	*gl2*/+; *nrpd2a*/+	*lfy*/+; *gl2*/+; *nrpd2a*/*nrpd2a*	WT: 33	*lfy*: 15	48	1.67E-05
		*lfy*/+; *gl2*/+; +/+	WT: 48	*lfy*: 0	48	
*ag*/+; *rdr6*/+	*gl2*/+; *rdr6*/+	*ag*/+; *gl2*/+; *rdr6*/*rdr6*	WT: 42	*ag*: 0	42	1
		*ag*/+; *gl2*/+; +/+	WT: 47	*ag*: 0	47	
*lfy*/+; *rdr6*/+	*lfy*/+; *rdr6*/+	*lfy*/+; *gl2*/+; *rdr6*/*rdr6*	WT: 43	*lfy*: 0	43	1
		*lfy*/+; *gl2*/+; +/*+*	WT: 46	*lfy*: 0	46	

*Fisher’s exact test.
