# Supplementary material for: Epigenetic Regulation of Intronic Transgenes in Arabidopsis
Source: Sci Rep. 2017 Mar 24;7:45166. doi: 10.1038/srep45166 (PMC5364540; doi:10.1038/srep45166)
Supplement: Supplementary Figures [file srep45166-s1.pdf]

Supplementary Information

Title: Epigenetic Regulation of Intronic Transgenes in *Arabidopsis*

Authors: Kenji Osabe, Yoshiko Harukawa, Saori Miura, and Hidetoshi Saze.

Supplementary Figs. S1-S11.

Supplementary Table S1.

Figure S1

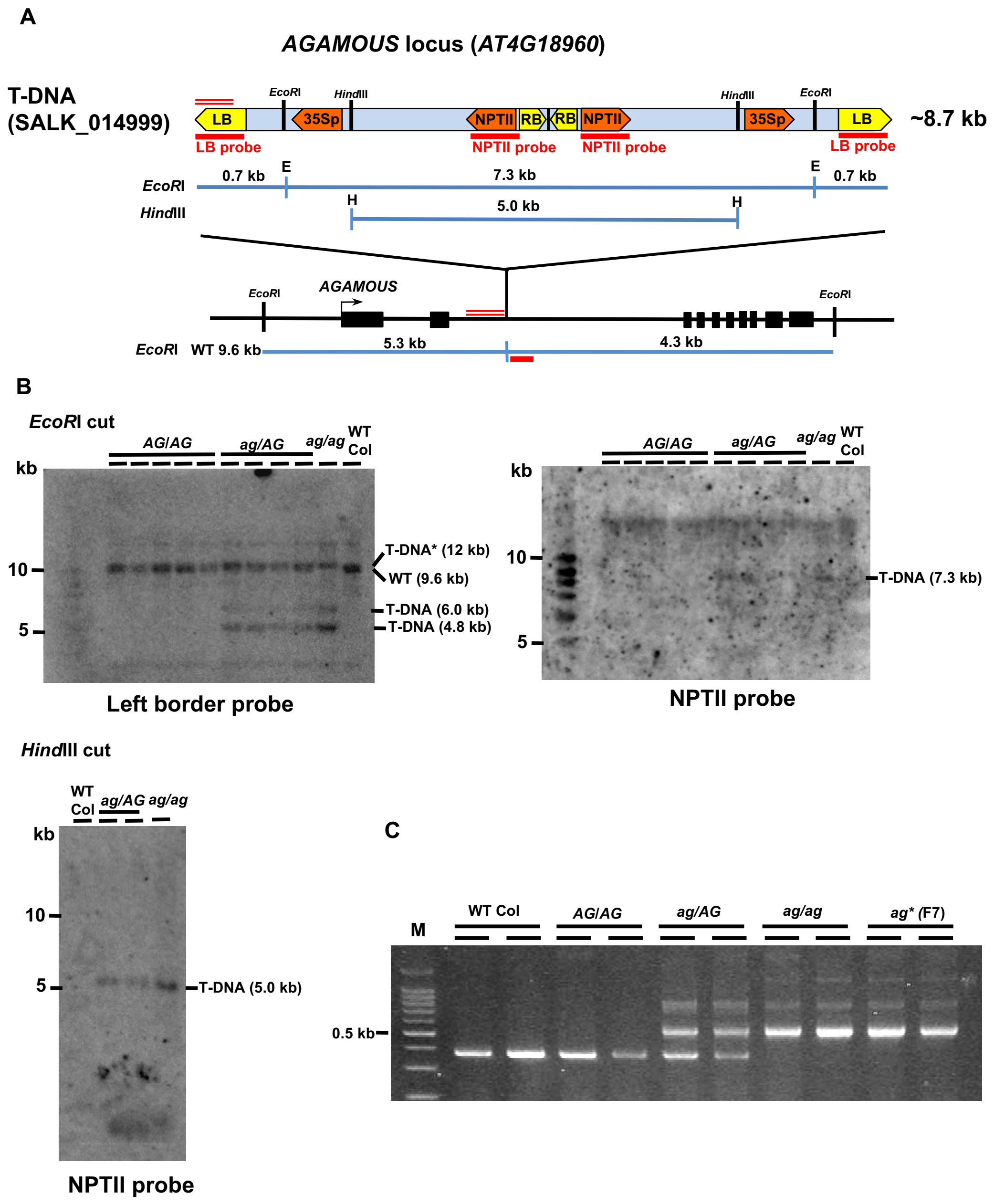

Supplementary Figure 1. Gene and T-DNA structures inserted into the *AGAMOUS* locus. (A) T-DNA sequences determined by sequencing analysis, and expected sizes of DNA fragments after digestion with *Eco*RI and *Hind*III. The red, double bar represents the 5' junction of the T-DNA insertion site analysed by bisulfite-sequencing. (B) Southern analysis of DNAs from wild-type Columbia (WT Col) and segregating progen of *ag* T-DNA mutant. DNAs were digested with either *Eco*RI (upper two panels) or *Hind*III (the lower panel) and hybridised with probes indicated in (A). Note that the left border probe contained part of a flanking sequence from the *AG* locus and therefore detected a wild-type DNA fragment from the *AG* locus. * represents a band of unknown origin. (C) A gel image of PCR-genotyping for *AG*. M: 100bp DNA ladder marker.

Figure S2

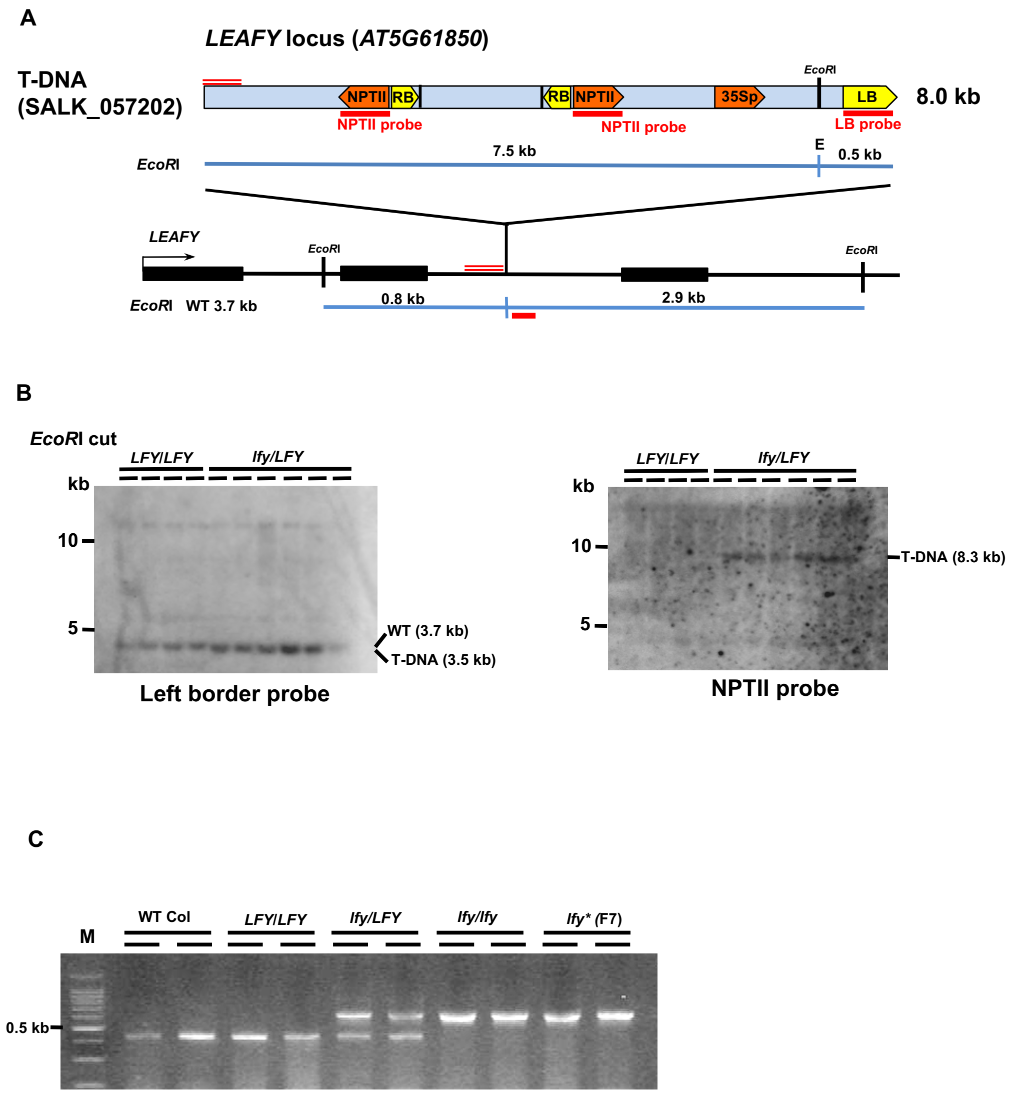

Supplementary Figure 2. Gene and T-DNA structures inserted into the *LEAFY* locus. (A) A T-DNA sequence determined by sequencing analysis, and expected sizes of DNA fragments after digestion with *EcoR*I. The double bar represents the 5' junction of the T-DNA insertion site analysed by bisulfite-sequencing. (B) Southern analysis of DNAs from wild-type Columbia (WT Col) and segregating progeny of a *lfy* T-DNA mutant. DNAs were digested with *EcoR*I and hybridised with probes indicated in (A). Note that the left border probe contained part of the flanking sequence from the *lfy* locus; therefore, it detected a wild-type DNA fragment from the *LFY* locus. (C) A gel image of PCR-genotyping for *LFY*. M: 100bp DNA ladder marker.

Figure S3

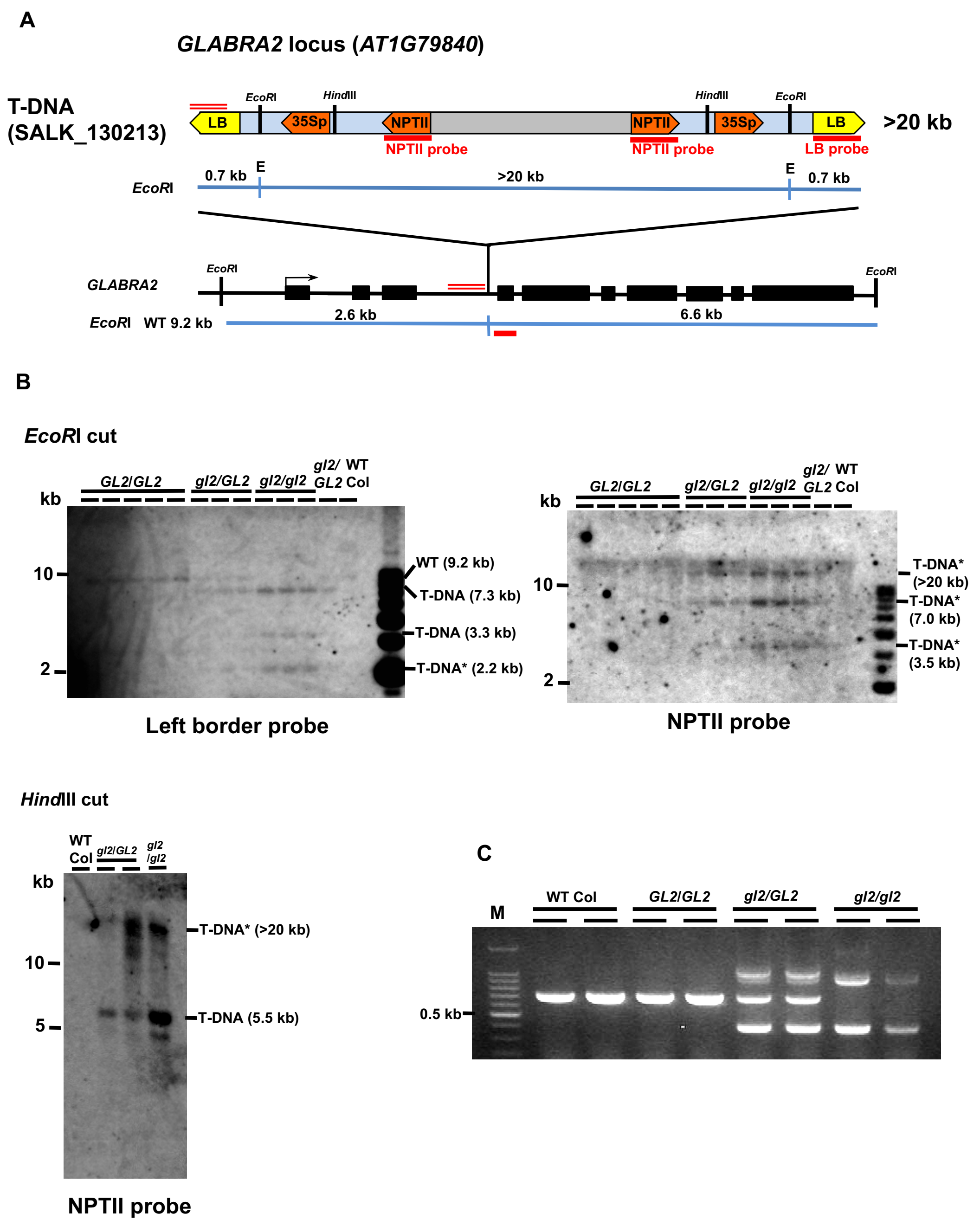

Supplementary Figure 3. Gene and T-DNA structures inserted into the *GLABRA2* locus. (A) T-DNA sequence determined by sequencing analysis, and expected sizes of DNA fragments after digestion with *Eco*RI. The gray box represents a sequence that was not determined by sequencing analysis. The double bar represents the 5′ junction of the T-DNA insertion site analysed by bisulfite-sequencing. (B) Southern analysis of DNAs from wild-type Columbia (WT Col) and segregating progeny of *gl2* T-DNA mutant. DNAs were digested with either *Eco*RI (upper two panels) or *Hind*III (lower panel) and hybridised with probes indicated in (A). Note that the left border probe contained part of the flanking sequence from the *GL2* locus; therefore it detected a wild-type DNA fragment from the *GL2* locus. * represents bands of unknown origin. (C) A gel image of PCR-genotyping for*GL2*. M: 100bp DNA ladder marker.

Figure S4

A

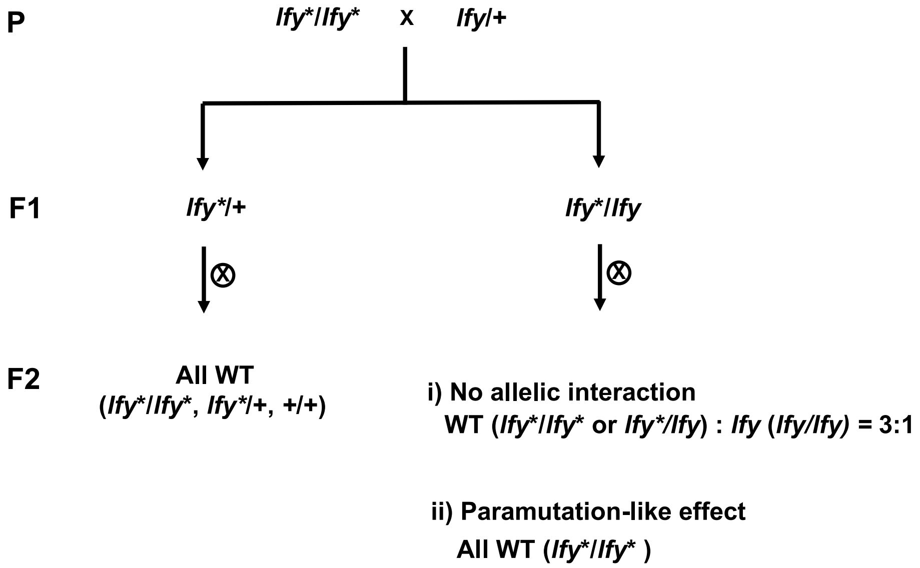

B

Cross	F1 (epi) genotype	F2 phenotype	*n*	*p-value**
lfy/lfy** #1 x *lfy/+*	*lfy*/+* #1-1	WT: 34 *lfy*: 0	34	-
	lfy/+* #1-2	WT: 33 *lfy*: 0	33	-
	lfy/lfy* #1-1	WT: 27 *lfy*: 0	27	0.0027
	lfy/lfy* #1-2	WT: 25 *lfy*: 0	25	0.0039
lfy/lfy** #2 x *lfy/+*	*lfy*/+* #2-1	WT: 29 *lfy*: 0	29	-
	lfy/+* #2-2	WT: 31 *lfy*: 0	31	-
	lfy/lfy* #2-1	WT: 33 *lfy*: 0	33	0.0009
	lfy/lfy* #2-2	WT: 31 *lfy*: 0	31	0.0013

***Chi-square test with the expectation of 3:1 segregation ratio.**

Supplementary Figure 4. A paramutation-like effect of the suppressed *lfy** allele. (A) Crossing scheme of *lfy* and *lfy** mutants. (B) A summary table of the *lfy* phenotyping in the F2 population. Results from two independent crosses are shown.

Figure S5

A

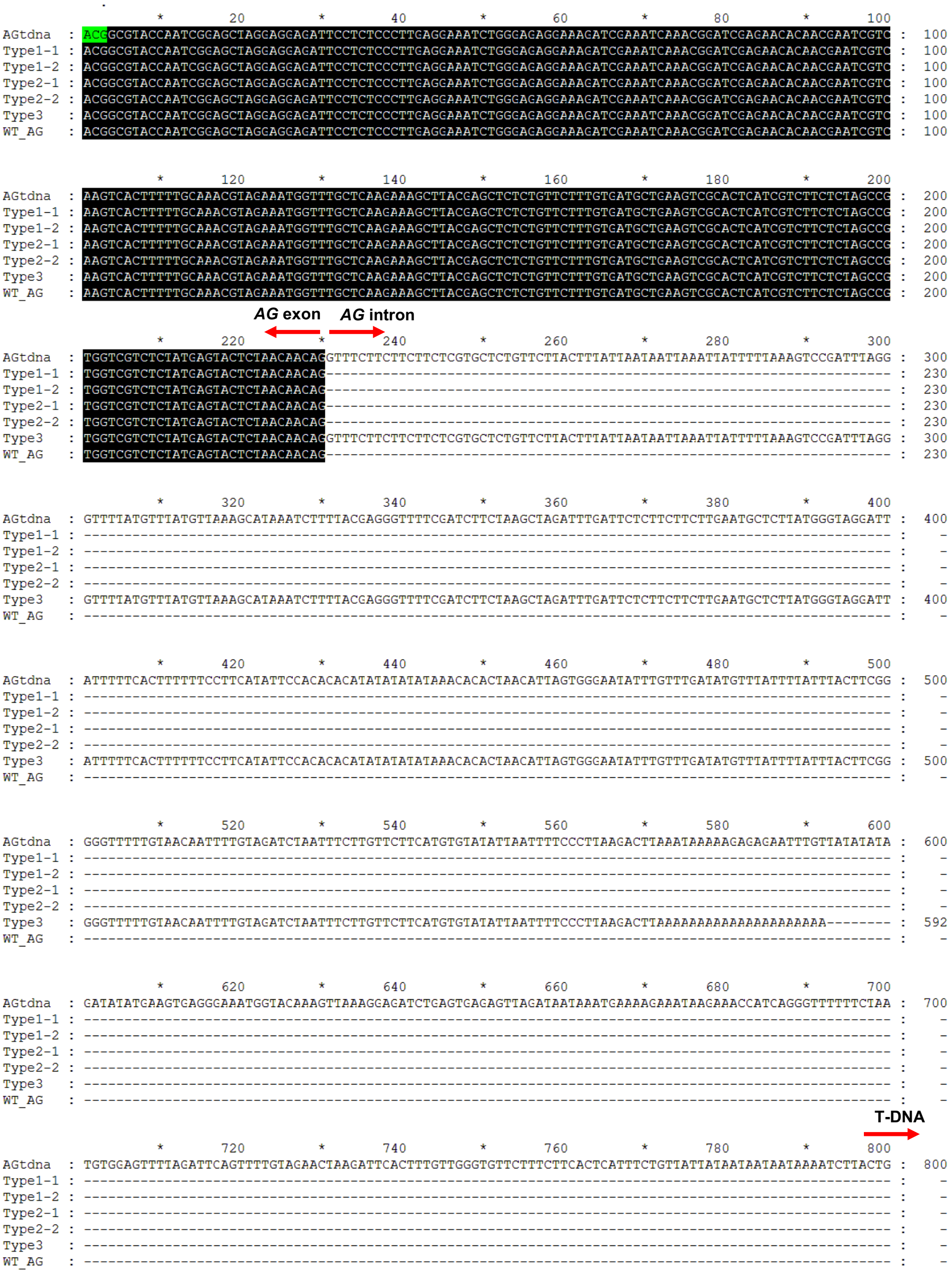
AGtdna
Type1-1
Type1-2
Type2-1
Type2-2
Type3
WT_AG
AG exon
AG intron
T-DNA

Figure S5 (continued)

```
                T-DNA
                      *        820         *        840         *        860         *        880         *        900
AGtdna   : ATGGGCTGCCTGTATCGAGTGGTGATTTTGTGCCGAGCTGCCGGTCGGGGAGCTGTTGGCTGGCTGGTGGCAGGATATATTGTGGTGTAAACAAATTGAC :  900
Type1-1  : ---------------------------------------------------------------------------------------------------- :    -
Type1-2  : -------------------------------------------------------------------------GATATATTGTGGTGTAAACAAATTGAC :  257
Type2-1  : ---------------------------------------------------------------------------------------------------- :    -
Type2-2  : -------------------------------------------------------------------------GATATATTGTGGTGTAAACAAATTGAC :  257
Type3    : ---------------------------------------------------------------------------------------------------- :    -
WT_AG    : ---------------------------------------------------------------------------------------------------- :    -

                      *        920         *        940         *        960         *        980         *       1000
AGtdna   : GCTTAGACAACTTAATAACACATTGCGGACGTTTTTAATGTACTGGGGTGGTTTTTCTTTTCACCAGTGAGACGGGCAACAGCTGATTGCCCTTCACCGC : 1000
Type1-1  : -------------------------------------------------------------------TGAGACGGGCAACAGCTGATTGCCCTTCACCGC :  263
Type1-2  : GCTTAGACAACTTAATAACACATTGCGGACGTTTTTAATGTACTGGGGTGGTTTTTCTTTTCACCAGTGAGACGGGCAACAGCTGATTGCCCTTCACCGC :  357
Type2-1  : -------------------------------------------------------------------TGAGACGGGCAACAGCTGATTGCCCTTCACCGC :  263
Type2-2  : GCTTAGACAACTTAATAACACATTGCGGACGTTTTTAATGTACTGGGGTGGTTTTTCTTTTCACCAGTGAGACGGGCAACAGCTGATTGCCCTTCACCGC :  357
Type3    : ---------------------------------------------------------------------------------------------------- :    -
WT_AG    : ---------------------------------------------------------------------------------------------------- :    -

                      *       1020         *       1040         *       1060         *       1080         *       1100
AGtdna   : CTGGCCCTGAGAGAGTTGCAGCAAGCGGTCCACGCTGGTTTGCCCCAGCAGGCGAAAATCCTGTTTGATGGTGGTTCCGAAATCGGCAAAATCCCTTATA : 1100
Type1-1  : CTGGCCCTGAGAGAGTTGCAGCAAGCGGTCCACGCTGGTTTGCCCCAGCAGGCGAAAATCCTGTTTGATGGTGGTTCCGAAATCGGCAAAATCCCTTATA :  363
Type1-2  : CTGGCCCTGAGAGAGTTGCAGCAAGCGGTCCACGCTGGTTTGCCCCAGCAGGCGAAAATCCTGTTTGATGGTGGTTCCGAAATCGGCAAAATCCCTTATA :  457
Type2-1  : CTGGCCCTGAGAGAGTTGCAGCAAGCGGTCCACGCTGGTTTGCCCCAGCAGGCGAAAATCCTGTTTGATGGTGGTTCCGAAATCGGCAAAATCCCTTATA :  363
Type2-2  : CTGGCCCTGAGAGAGTTGCAGCAAGCGGTCCACGCTGGTTTGCCCCAGCAGGCGAAAATCCTGTTTGATGGTGGTTCCGAAATCGGCAAAATCCCTTATA :  457
Type3    : ---------------------------------------------------------------------------------------------------- :    -
WT_AG    : ---------------------------------------------------------------------------------------------------- :    -

                      *       1120         *       1140         *       1160         *       1180         *       1200
AGtdna   : AATCAAAAGAATAGCCCGAGATAGGGTTGAGTGTTGTTCCAGTTTGGAACAAGAGTCCACTATTAAAGAACGTGGACTCCAACGTCAAAGGGCGAAAAAC : 1200
Type1-1  : AAAAAAAAAAAAAA-------------------------------------------------------------------------------------- :  377
Type1-2  : AAAAAAAAAAAAAAAA------------------------------------------------------------------------------------ :  473
Type2-1  : AATCAAAAGAATAGCCCGAGATAGGGTTGAGTGTTGTTCCAGTTTGGAACAAGAGTCCACTATTAAAGAACGTGGACTCCAACGTCAAAGGGCGAAAAAC :  463
Type2-2  : AATCAAAAGAATAGCCCGAGATAGGGTTGAGTGTTGTTCCAGTTTGGAACAAGAGTCCACTATTAAAGAACGTGGACTCCAACGTCAAAGGGCGAAAAAC :  557
Type3    : ---------------------------------------------------------------------------------------------------- :    -
WT_AG    : ---------------------------------------------------------------------------------------------------- :    -

                      *       1220         *       1240         *       1260         *       1280         *       1300
AGtdna   : CGTCTATCAGGGCGATGGCCCACTACGTGAACCATCACCCAAATCAAGTTTTTTGGGGTCGAGGTGCCGTAAAGCACTAAATCGGAACCCTAAAGGGAGC : 1300
Type1-1  : ---------------------------------------------------------------------------------------------------- :    -
Type1-2  : ---------------------------------------------------------------------------------------------------- :    -
Type2-1  : CGTCTATCAGGGCGATGGCCCACTACGTGAACCATCACCCAAATCAAGTTTTTTGGGGTCGAGGTGCCGTAAAGCACTAAATCGGAACCCTAAAGGGAGC :  563
Type2-2  : CGTCTATCAGGGCGATGGCCCACTACGTGAACCATCACCCAAATCAAGTTTTTTGGGGTCGAGGTGCCGTAAAGCACTAAATCGGAACCCTAAAGGGAGC :  657
Type3    : ---------------------------------------------------------------------------------------------------- :    -
WT_AG    : ---------------------------------------------------------------------------------------------------- :    -

                      *       1320         *       1340         *       1360         *       1380         *       1400
AGtdna   : CCCCGATTTAGAGCTTGACGGGGAAAGCCGGCGAACGTGGCGAGAAAGGAAGGAAGAAAGCGAAAGGAGCGGGCGCCATTCAGGCTGCGCAACTGTTGG : 1400
Type1-1  : ---------------------------------------------------------------------------------------------------- :    -
Type1-2  : ---------------------------------------------------------------------------------------------------- :    -
Type2-1  : CCCCGATTTAGAGCTTGACGGGGAAAGCCGGCGAACGTGGCGAGAAAGGAAGGAAGAAAGCGAAAGGAGCGGGCGCCATTCAGGCTGCGCAACTGTTGG :  663
Type2-2  : CCCCGATTTAGAGCTTGACGGGGAAAGCCGGCGAACGTGGCGAGAAAGGAGGGAAGAAAGCGAAAGGAGCGGGCGCCATTCAGGCTGCGCAACTGTTGG :  757
Type3    : ---------------------------------------------------------------------------------------------------- :    -
WT_AG    : ---------------------------------------------------------------------------------------------------- :    -

                      *       1420         *       1440         *       1460         *       1480         *       1500
AGtdna   : GAAGGGCGATCGGTGCGGGCCTCTTCGCTATTACGCCAGCTGGCGAAAGGGGGATGTGCTGCAAGGCGATTAAGTTGGGTAACGCCAGGGTTTTCCCAGT : 1500
Type1-1  : ---------------------------------------------------------------------------------------------------- :    -
Type1-2  : ---------------------------------------------------------------------------------------------------- :    -
Type2-1  : GAAGGGCGATCGGTGCGGGCCTCTTCGCTATTACGCCAGCTGGCGAAAGGGGGATGTGCTGCAAGGCGATTAAGTTGGGTAACGCCAGGGTTTTCCCAGT :  763
Type2-2  : GAAGGGCGATCGGTGCGGGCCTCTTCGCTATTACGCCAGCTGGCGAAAGGGGGATGTGCTGCAAGGCGATTAAGTTGGGTAACGCCAGGGTTTTCCCAGT :  857
Type3    : ---------------------------------------------------------------------------------------------------- :    -
WT_AG    : ---------------------------------------------------------------------------------------------------- :    -

                      *       1520         *       1540         *       1560         *       1580         *       1600
AGtdna   : CACGACGTTGTAAAACGACGGCCAGTGAATTCCCGATCTAGTAACATAGATGACACCGCGCGCGATAATTTATCCTAGTTTGCGCGCTATATTTTGTTTT : 1600
Type1-1  : ---------------------------------------------------------------------------------------------------- :    -
Type1-2  : ---------------------------------------------------------------------------------------------------- :    -
Type2-1  : CACGACGTTGTAAAACGACGGCCAGTGAATTCCCGATCTAGTAACATAGATGACACCGCGCGCGATAATTTATCCTAGTTTGCGCGCTATATTTTGTTTT :  863
Type2-2  : CACGACGTTGTAAAACGACNGCCAGTGAATTCCCGATCTAGTAACATAGATGACACCGCGCGCGATAATTTATCCTAGTTTGCGCGCTATATTTTGTTTT :  957
Type3    : ---------------------------------------------------------------------------------------------------- :    -
WT_AG    : ---------------------------------------------------------------------------------------------------- :    -
```

Figure S5 (continued)

T-DNA

```
                 *      1620        *      1640        *      1660        *      1680        *      1700
AGtdna  : CTATCGCGTATTAAATGTATAATTGCGGGACTCTAATCATAAAAACCCATCTCATAAATAACGTCATGCATTACATGTTAATTATTACATGCTTAACGTA : 1700
Type1-1 : ---------------------------------------------------------------------------------------------------- :    -
Type1-2 : ---------------------------------------------------------------------------------------------------- :    -
Type2-1 : CTATCGCGTATTAAATGTATAATTGCGGGACTCTAATCATAAAAACCCATCTCATAAAAAAAAAAAAAAAAAAAAA------------------------- :  938
Type2-2 : CTATCGCGTATTAAATGTATAATTGCGGGACTCTAATCATAAAAACCCATCTCATAAATAAAAAAAAAAAAAAAAAA----------------------- : 1034
Type3   : ---------------------------------------------------------------------------------------------------- :    -
WT_AG   : ---------------------------------------------------------------------------------------------------- :    -
```

B

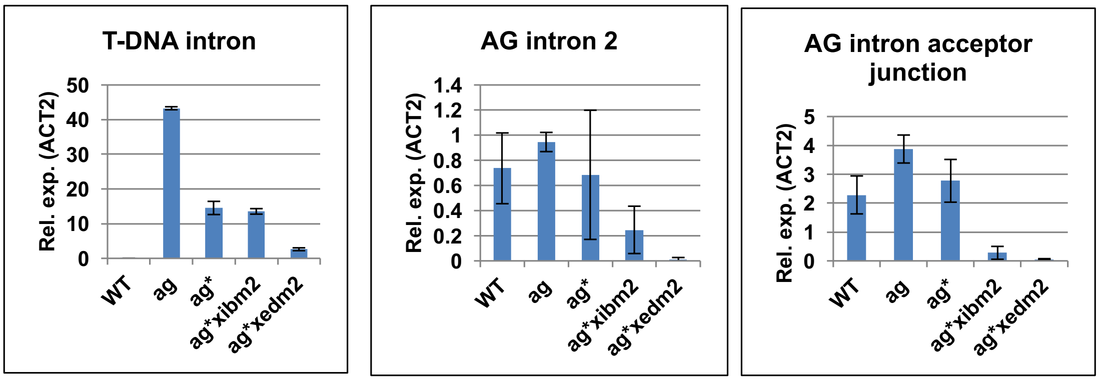

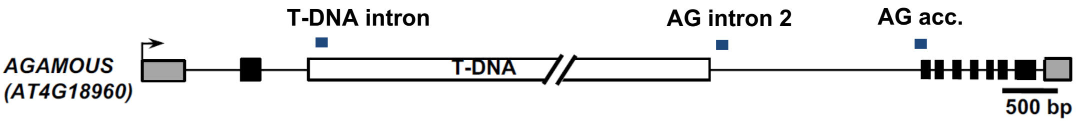

Supplementary Figure 5. (A) Transcript isoforms of *AGAMOUS* (*AG*) containing introns or T-DNA sequences identified by sequencing of RACE products from *ag*, *ag**, *ag*ibm2*, and *ag*edm2*. Sequences are aligned against the WT AG genomic DNA sequence from the start codons. Black shading represents the exon sequence of *AG*. Types 1-1 and 1-2 share similar polyadenylation sites, but have different acceptor sites. Types 2-1 and 2-2 share similar polyadenylation sites and share the same splice acceptor site as Types1-1 and 1-2, respectively. (B) *AG* expression level measured by qRT-PCR. *ag*; parental *ag* mutant, *ag**; suppressed *ag*, WT; non-transgenic Columbia. Bars are mean +/- SEM (n=3).

Figure S6

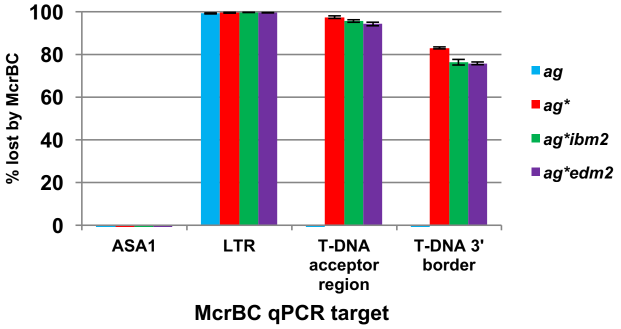

Supplementary Figure 6. McrBC-qPCR of *ag*, *ag**, *ag*ibm2*, and *ag*edm2* to measure the level of DNA methylation of T-DNA regions. Error bars represent the standard errors of means from four biological replicates of each line. *ASA1* and LTR regions have been selected as unmethylated and methylated controls for the McrBC digest, respectively. The *ASA1* region was not digested and the LTR shows almost complete digestion by McrBC, as expected. Both the T-DNA splice acceptor site (approximately 100 bp flanking the acceptor site) and the 3′ border region are unmethylated in *ag*, but heavily methylated in *ag**, *ag*ibm2*, and *ag*edm2*.

Figure S7

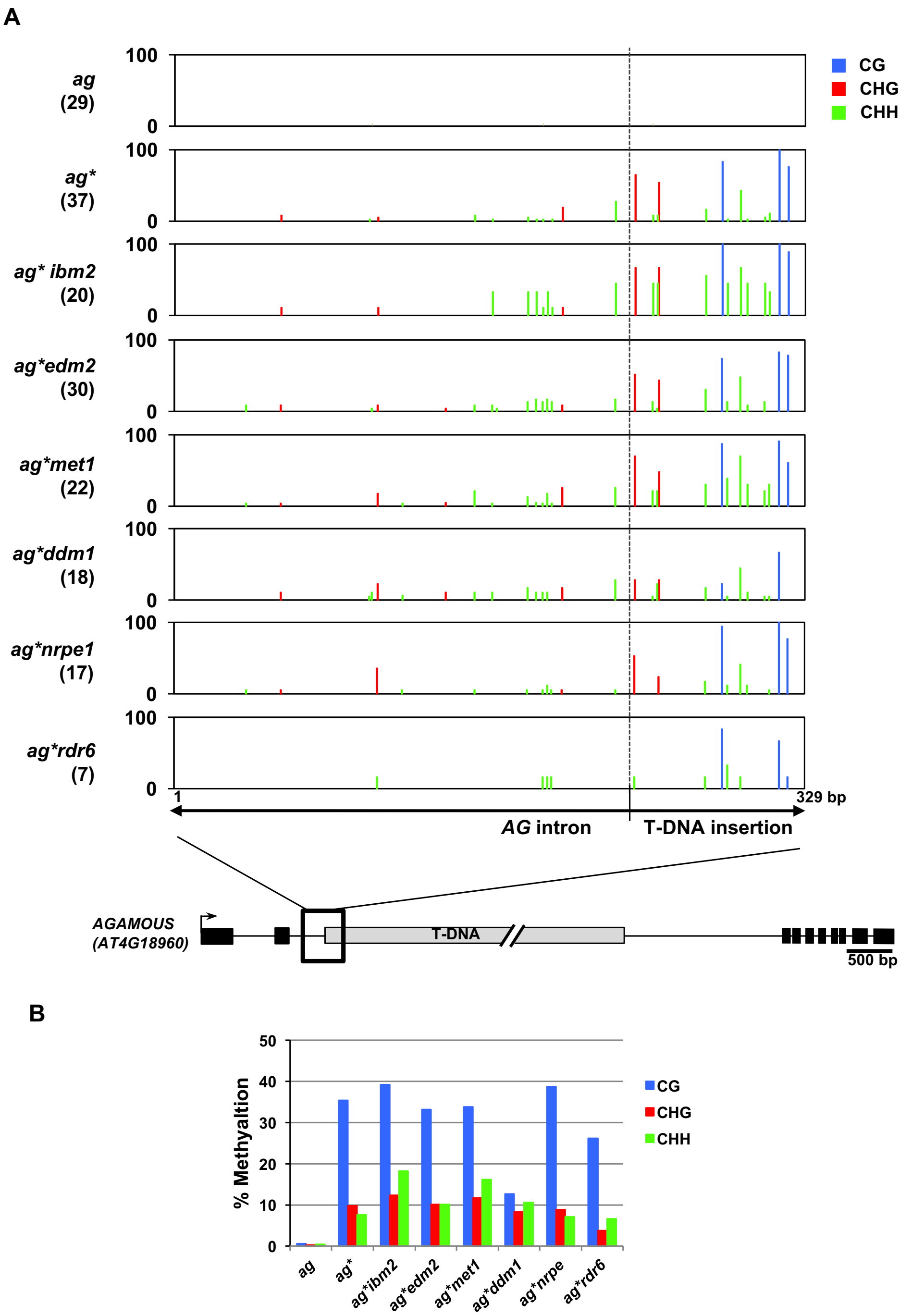

Supplementary Figure 7. BS-seq of the 5′ border of the T-DNA insertion site and the flanking *AG* intron. (A) A graphical representation of the DNA methylation status (CG, CHG, and CHH) of representative samples with indicated genotypes. Numbers in parentheses indicate the number of independent clones sequenced for each genotype. The dotted line represents the border between the *AG* intron and T-DNA insertion. (B) DNA methylation context-dependent summary of samples analysed in (A).

Figure S8

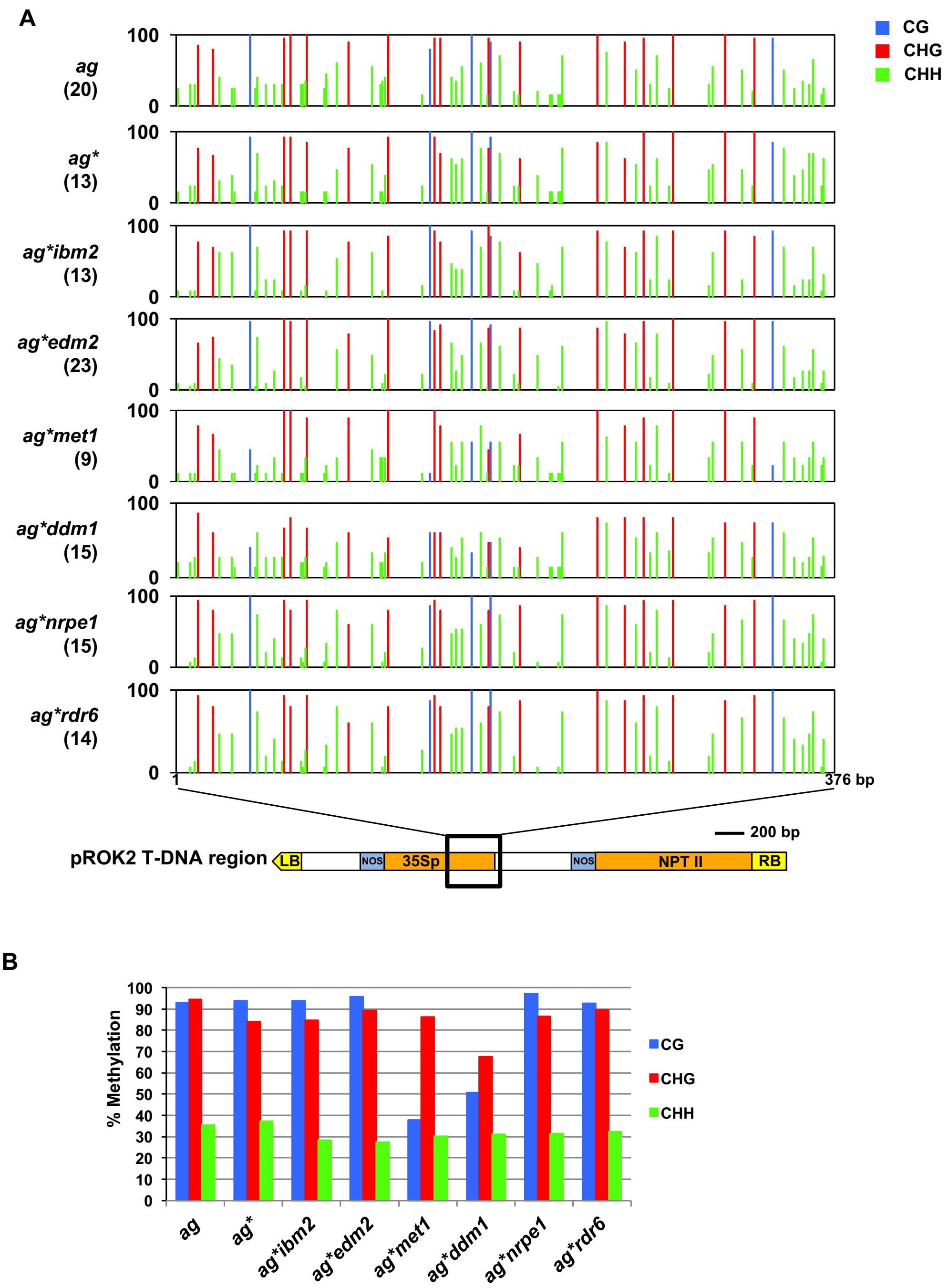

Supplementary Figure 8. BS-seq of the 35S promoter sequence of T-DNA inserted in the *AGAMOUS* intron. (A) A graphical representation of the DNA methylation status (CG, CHG, and CHH) of representative samples with indicated genotypes. Numbers in parentheses indicate the number of independent clones sequenced for each genotype. Note that at least two 35S promoter sequences are found in the T-DNA sequence inserted into the *AG* locus; therefore, results are from mixed PCR products for these sequences. (B) DNA methylation context-dependent summary of samples analysed in (A).

Figure S9

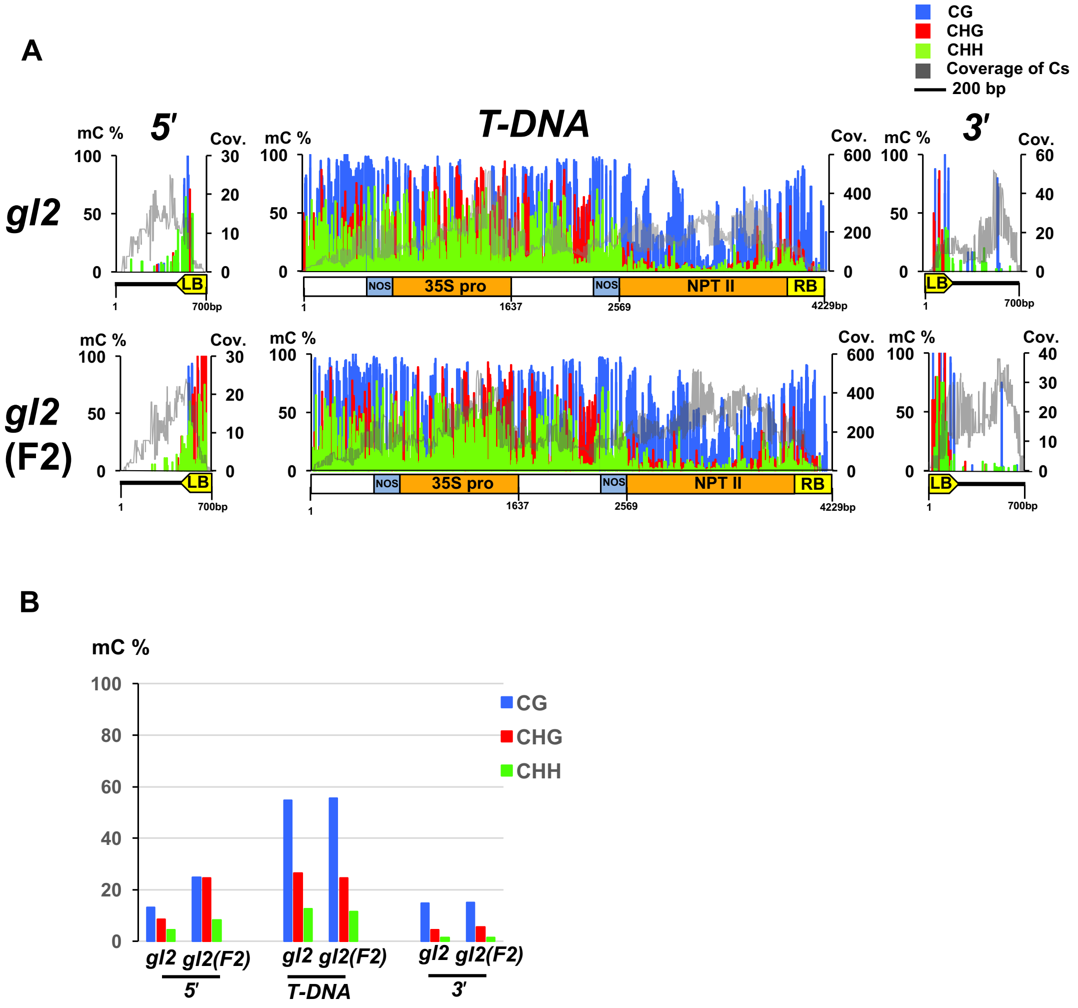

Supplementary Figure 9. BS-seq analyses of the 5′ and 3′ borders of the T-DNA insertion site and flanking intron sequences, as well as T-DNA regions in *gl2* single mutants. (A) A graphical representation of DNA methylation status (CG, CHG, and CHH) of representative samples with the indicated genotypes/epigenotypes. *gl2* represents DNA methylation in the T-DNA regions of T4 homozygous plants before crossing. For *gl2* (F2), genomic DNA from *gl2* single mutants after crossing to *ag* plants was used, in which the absence of T-DNA in the *ag* locus was confirmed by PCR. Gray lines represent the number of cytosines covered by BS-seq reads. Cytosines that covered fewer than 4 reads were excluded from the analysis. (B) A summary of DNA methylation analysed in (A).

Figure S10

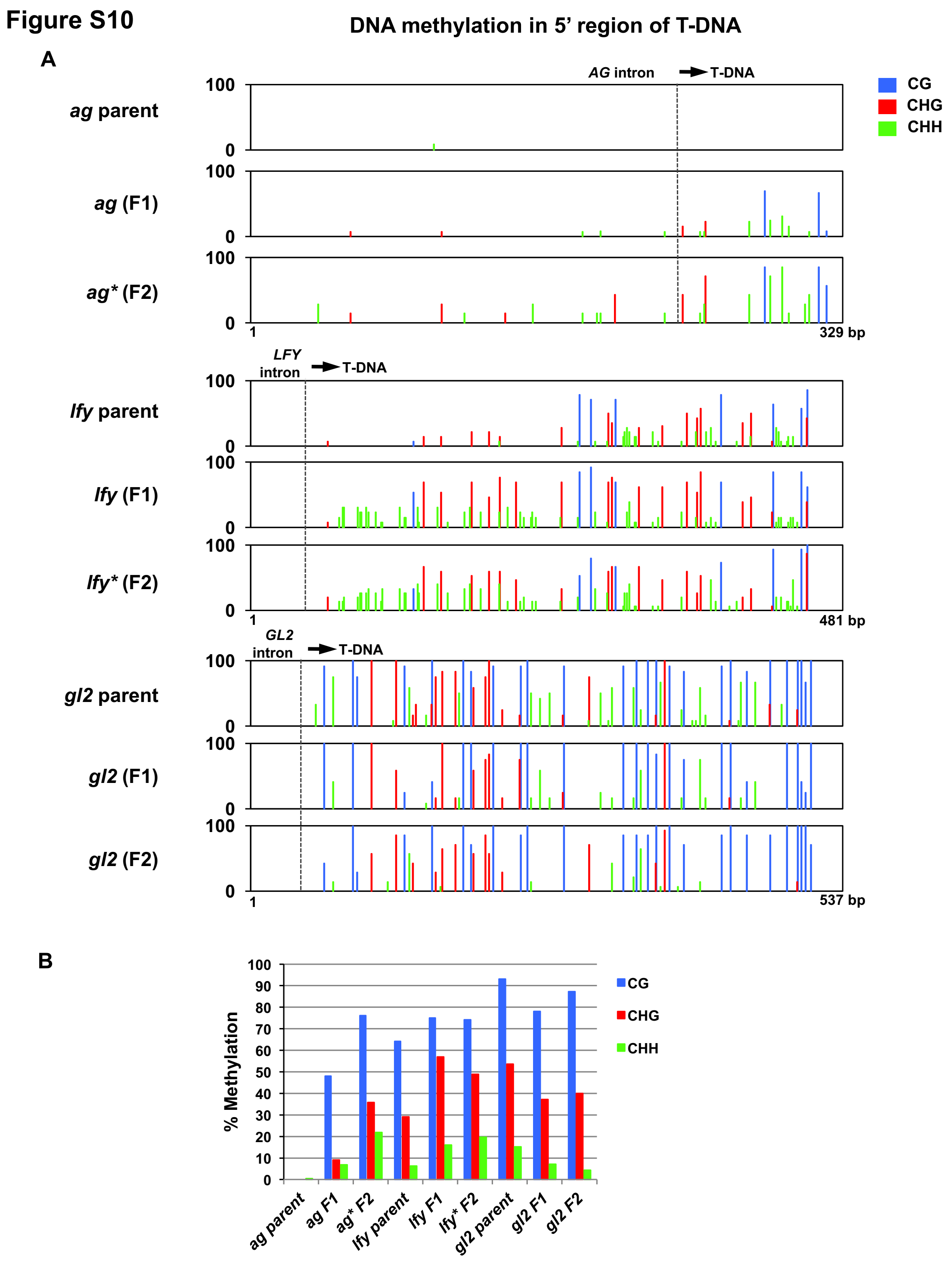

Supplementary Figure 10. BS-PCR of the 5′ border sequence of T-DNA inserted in the *AGAMOUS*, *LEAFY* and *GLABRA2* loci. (A) A graphical representation of DNA methylation status (CG, CHG, and CHH) of representative samples with the indicated genotypes. Ten to sixteen clones were sequenced for each genotype. 5′-flanking regions analysed by BS-seq were indicated in Supplementary Fig. S1-S3. (B) DNA methylation context-dependent summary of samples analysed in (A).

Figure S11

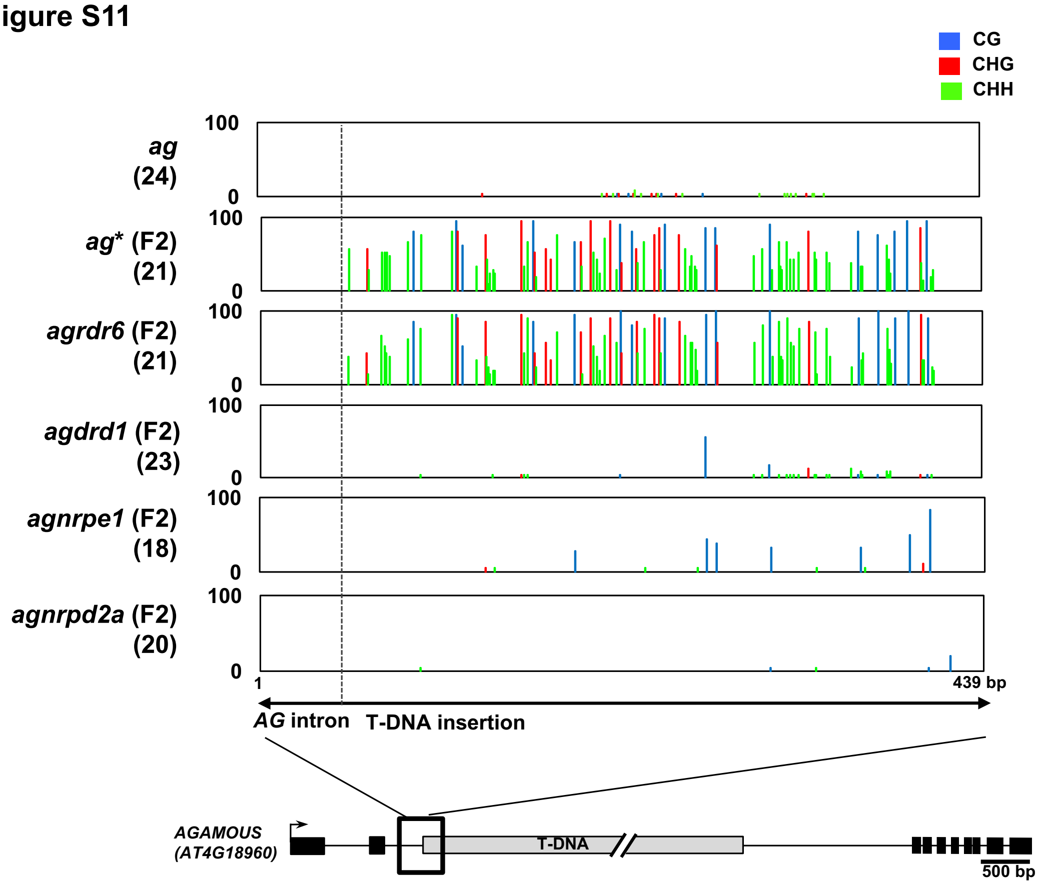

Supplementary Figure S11. BS-seq of the 5′ border of the T-DNA insertion site and the flanking *AG* intron. A graphical representation of DNA methylation status (CG, CHG, and CHH) of representative samples with indicated genotypes. Numbers in parentheses indicate the number of independent clones sequenced for each genotype. The dotted line represents the border between the *AG* intron and T-DNA insertion. For visualisation, DNA methylation status in the reverse strand is shown. A summary graph is shown in Fig. 5D.

Supplementary Table S1. Primers used in this study.

Target	Primers	Sequence (5' to 3')	Application
AGAMOUS	AG F1	TATGAAGTGAGGGAAATGGTAC	Genotyping
	AG R3	ACAATGGAGGATGGATGATCAC	Genotyping and McrBC qPCR of T-DNA 3'border
	LBa1	TGGTTCACGTAGTGGGCCATCG	Genotyping and McrBC qPCR of T-DNA 3'border
	KO293	TTYYYTTAAGAYTTAAATAAAAAGAGAGAATTTGT	BS-PCR (5' region of AG T-DNA: Fig. S7, S10)
	KO294	TRRTRAAAARAAAAACCACCCCARTAC	BS-PCR (5' region of AG T-DNA: Fig. S7, S10)
	35S BS F1	GTATGTTGTGTGGAATTGTGAGYGGATAA	BS-PCR (AG T-DNA: Fig. S8)
	35S BS R1	CCTTTTTARARACTCCAATCTCTATTACTT	BS-PCR (AG T-DNA: Fig. S7, S10)
	T-DNA LB Bs R2	TTGATTTGGGTGATGGTTYAYGTAGTGGG	BS-PCR (AG T-DNA: Fig. 5, S11)
	Ag int BS F2	TTCTTTCTTCACTCATTTCTRTTATTATAA	BS-PCR (AG T-DNA: Fig. 5, S11)
	KO085	ACGGCGTACCAATCG	3' RACE region 1 outer primer
	AGRTF1	ATCGGAGCTAGGAGGAGATTCC	3' RACE region 1 inner primer
	AGF1	TATGAAGTGAGGGAAATGGTAC	3' RACE region 2 outer primer
	KO104	ATGGTACAAAGTTAAAGGAGATC	3' RACE region 2 inner primer
	KO098	CGTCATCACTCAGATATTATTTC	3' RACE region 3 outer primer
	KO105	AGATATTATTTCTTTTTATTTTTCACTTG	3' RACE region 3 inner primer
	KO099	ACAAGAATCAGCCAAATTG	3' RACE region 4 outer primer
	KO106	TGCGTCAACAAATAATCAG	3' RACE region 4 inner primer
	KO101	GTCGCCAAGACCAAAC	3' RACE region 5 outer primer
	KO108	AAACCGCTCTCCAGTTAG	3' RACE region 5 inner primer
	KO158	GCCGTGGTCGTCTCTATGAG	qRT-PCR of AG exon1-T-DNA
	Lbc1	TAAGGGATTTTGCCGATTTCGGA	qRT-PCR of AG exon1-T-DNA
	KO123	CCAAACCGCTCTCCAGTTAG	qRT-PCR of AG 3' CDS
	KO124	GGCCATTTCCTTCAGCCTAT	qRT-PCR of AG 3' CDS
	KO156	GGGGAGAGAGTAAGGAAGGACT	qRT-PCR of AG intron
	KO157	ACTCTCACTTACCATCACATGTGT	qRT-PCR of AG intron
	KO160	TCAAAATGCAGATTTAAGCGTAGA	qRT-PCR of AG intron acceptor junction
	KO161	TCCGGTGTTAGAATTGTCCGA	qRT-PCR of AG intron acceptor junction
	KO259	AGGACTAGCCCAACCTTCAC	McrBC qPCR of ASA1
	KO260	GATCCCGACGGTGGTGAATT	McrBC qPCR of ASA1
	KO255	TTTGCTCTCAAACTCTCAATTGAAGTTT	McrBC qPCR of LTR
	KO256	TAGGGTTCTTAGTTGATCTTGTATTGAGCTC	McrBC qPCR of LTR
	KO262	TTGCTGCAACTCTCTCAGGG	McrBC qPCR of T-DNA acceptor site, qRT-PCR
	KO263	AACACATTGCGGACGTTTTT	McrBC qPCR of T-DNA acceptor site, qRT-PCR
LEAFY	LFY F3	CTATAGCTATAATCATGGACAG	Genotyping
	LFY R2	TCTGTACTATCATCACTAGAGG	Genotyping
	LBa1	TGGTTCACGTAGTGGGCCATCG	Genotyping
	LFY BS F1	AAAATTTAGGTTYTAATTTATTAAATTTT	BS-PCR (5' region of T-DNA insertion)
	35S BS C3 F2	CACCCCARRCTTTACACTTTATRCTTCC	BS-PCR (5' region of T-DNA insertion)
	LFY BS R1	CAAAGAAACAACTATGTCCTTTCCCTAACTC	BS-PCR (3' region of T-DNA insertion)
	LB BS G5 F2	TTGATTTGGGTGATGGTTYAYGTAGTGGG	BS-PCR (3' region of T-DNA insertion)
	KO125	ATTGGTTCAAGCACCACCTC	qRT-PCR of LFY upstream
	KO126	CAAGAAGCTCCCAACGAAAG	qRT-PCR of LFY upstream
	KO127	GGTACGCGAAGAAATCAGGA	qRT-PCR of LFY downstream

	KO128	ATGACGACAAGCGATGTTCA	qRT-PCR of LFY downstream
GRABLA2	GL2 F1	GTCACACCACCGATCAGATCAG	Genotyping
	GL2 R1	CTTGCTCAGCTGCTGTCTTTGC	Genotyping
	LBa1	TGGTTCACGTAGTGGGCCATCG	Genotyping
	GL2 BS F1	AGTTAGGGTTYAGTTGYATGTAAAGATTTT	BS-PCR (5' region of T-DNA insertion)
	LB BS C3 F1	CTCCTTTCRCTTTCTTCCCTTCCTTTCTC	BS-PCR (5' region of T-DNA insertion)
edm2-9	edm2-9 XbaI P1	GTAGTATCTGACTGTTTATATTTTTGGAATTATGTC	Genotyping (XbaI digestion after amplification)
	edm2-9 P2	CCATATAAGCACATATGATGAC	Genotyping (XbaI digestion after amplification)
Universal	oligodT T7 2-3	CAGTGAATTGTAATACGACTCACTATAGGNVTTTTT	3'-RACE
Outer adaptor	T7 Primer3	CAGTGAATTGTAATACGACTC	3'-RACE
Inner adaptor	T7	TAATACGACTCACTATAGGG	3'-RACE and sequencing
pGEM-T Easy	M13REV	CAGGAAACAGCTATGAC	Sequencing
